# Reassessing the Use of Race in Clinical Algorithms: An Interactive, Case-Based Session for Medical Students Using eGFR

**DOI:** 10.15766/mep_2374-8265.11412

**Published:** 2024-06-21

**Authors:** Vijayvardhan Kamalumpundi, Carolina Gonzalez Bravo, Ariele Andalon, Amy L. Conrad, Joyce Goins-Fernandez

**Affiliations:** 1 First-Year Internal Medicine Resident, Department of Medicine, Mayo Clinic; 2 Third-Year Medical Student, University of Iowa Roy J. and Lucille A. Carver College of Medicine; 3 Third-Year Physician Assistant Student, University of Iowa Roy J. and Lucille A. Carver College of Medicine; 4 Associate Professor of Pediatrics, Division of Pediatric Psychology, Stead Family Department of Pediatrics, University of Iowa Roy J. and Lucille A. Carver College of Medicine; 5 Clinical Associate Professor of Pediatrics, Division of Pediatric Psychology, Stead Family Department of Pediatrics, University of Iowa Stead Family Children's Hospital; Interim Associate Dean of Health Parity, University of Iowa Roy J. and Lucille A. Carver College of Medicine; † Co-primary authors

**Keywords:** Clinical Algorithms, Clinical Decision-Making, Health Outcomes, Kidney Transplant, Case-Based Learning, Clinical Reasoning/Diagnostic Reasoning, Health Disparities, Health Equity, Nephrology, Anti-racism, Diversity, Equity, Inclusion

## Abstract

**Introduction:**

Medical curricula implicitly teach that race has a biological basis. Clinical rotations reinforce this misconception as race-based algorithms are used to guide clinical decision-making. This module aims to expose the fallacy of race in clinical algorithms, using the estimated glomerular filtration rate (eGFR) equation as an example.

**Methods:**

We created a 60-minute module in consultation with nephrologists. The format was an interactive, case-based presentation with a didactic section. A third-year medical student facilitated the workshops to medical students. Evaluation included pre/post surveys using 5-point Likert scales to assess awareness regarding use of race as a biological construct. Higher scores indicated increased awareness.

**Results:**

Fifty-five students participated in the module. Pre/post results indicated that students significantly improved in self-perceived knowledge of the history of racism in medicine (2.6 vs. 3.2, *p* < .001), awareness of race in clinical algorithms (2.7 vs. 3.7, *p* < .001), impact of race-based eGFR on quality of life/treatment outcomes (4.5 vs. 4.8, *p* = .01), differences between race and ancestry (3.7 vs. 4.3, *p* < .001), and implications of not removing race from the eGFR equation (2.7 vs. 4.2, *p* < .001). Students rated the workshops highly for quality and clarity.

**Discussion:**

Our module expands on others’ work to expose the fallacy of race-based algorithms and define its impact on health equity. Limitations include a lack of objective assessment of knowledge acquisition. We recommend integrating this module into preclinical and clinical curricula to discuss the use of race in medical literature and clinical practice.

## Educational Objectives

By the end of this session, learners will be able to:

1. Discuss how race is used in clinical algorithms, with an emphasis on the estimated glomerular filtration rate (eGFR) equation.
2. Explain how race-based clinical algorithms can magnify existing inequalities in health care.
3. Identify differences between race, ancestry, and genetics.
4. Describe the implications of removing the race-correction factor from the eGFR equation.

## Introduction

Since the creation of racial hierarchies in the early 18th century, biomedical research and policy have been shaped by the influence of race.^1,2^ Throughout history, individuals wielding authority have employed a biological understanding of race to justify discriminatory policies and promote White superiority. Notably, former President of the United States Thomas Jefferson attributed dysfunction of Blacks’ “pulmonary apparatus” as a basis for their suitability for fieldwork.^3^ In 1851, Dr. Samuel Cartwright, a leading Confederate physician, asserted physiologic differences between Blacks and Whites, justifying Black servitude. In parallel, dissenting voices emerged through the early work of W. E. B. Du Bois and Kelly Miller, who mounted insightful criticisms of the medical literature, citing the lack of historical and social context in evidence supporting race-based medicine.^3^ In 1971, the Black Panther party launched a campaign exposing racial biases in the medical system's recognition and treatment of sickle cell disease, which predominantly affects people of African descent.^4^ The collective efforts of prominent Black scholars and targeted clinical outreach laid the groundwork for a more nuanced understanding of race and medicine/health. In the 21st century, the Black Lives Matter movement has compelled institutions to reexamine sociopolitical structures promoting the marginalization of Black and minority communities, including health care systems and medical education.^5^

Over time, our understanding of race has shifted from rigid categorizations to recognizing its complex role in the interplay of social, cultural, and genetic factors influencing health. Consequently, the use of race has become more intricate and multifaceted. In clinical practice, a biological understanding of race is used to explain differences in adverse outcomes among people of color and in the development of treatment guidelines and clinical algorithms.^6^ However, race as a variable in medical literature lacks consistent definition, with infrequent reporting on whether studies use a patient's self-reported race, ethnicity, or ancestry. Shanawani, Dame, Schwartz, and Cook-Deegan demonstrated that 72% of 268 published studies reporting associations between race or ethnicity, health outcome, and genotype failed to explain their method of assigning race or ethnicity as an independent variable.^7^ Race is a social construct with no genetic basis and is a poor proxy for biological variation, yet its use as a biological variable persists in clinical medicine through race correction. Race correction involves incorporating race into an algorithm to guide a health care provider in interpreting a specific test result.^8^ Race correction is common across medical specialties.^8–10^ A poignant example is the upward adjustment of estimated glomerular filtration rate (eGFR) for Black patients, based on unfounded assertions that Black people have a higher muscle composition as opposed to White people.^11,12^ This artificial increase in eGFR of Black patients has led to significant health disparities in kidney transplant referral rates and renal care.^13^ As a consequence, several medical institutions have removed race from the eGFR equation and from clinical calculators in other specialties.^8–10^

Currently, a substantial number of trainees and medical students harbor inaccurate beliefs about racial differences in medicine.^14,15^ Moreover, medical curricula frequently misrepresent race as a biologic variable. Amutah and colleagues identified five domains where a preclinical curriculum misrepresented race: semantics, disease prevalence without appropriate social context, race-based diagnostic bias, pathologizing race, and race-based clinical guidelines.^14^ Clinical rotations reinforce this misconception as race-based algorithms are used to guide clinical decision-making. A review of *MedEdPORTAL* revealed 231 results using the phrase *race in medicine* and 99 results using the key phrase *clinical algorithms.* The only educational innovation recently developed to appraise the use of race in medical research is the Critical Appraisal of Race in Medical Literature tool.^16^ However, this tool does not target medical students in the preclinical to clinical years of their education. Additionally, existing medical curricula that expose the use of race in clinical algorithms seldom integrate insights from diverse disciplines like public health, medical ethics, and epidemiology. This hinders students from developing a holistic understanding of the causes and consequences of using race to guide clinical decision-making, as well as the epidemiologic considerations for removing race-correction factors. To address this gap, we developed an interactive, case-based session to elucidate the fallacy of incorporating race into clinical algorithms, with a focus on the eGFR equation. Our educational innovation aimed to build upon existing efforts, working to counteract the misrepresentation of race as a biologic construct and to unveil the inherent flaws in race-based clinical algorithms.

## Methods

### Module Development and Implementation

We developed an educational module elucidating the fallacy of incorporating race into clinical algorithms, with an emphasis on the eGFR equation. We also highlighted the profound implications of race correction for health equity, epidemiologic considerations for removing race-correction factors, and crucial distinctions between race, ancestry, and genetics. This module was developed in consultation with specialists in adult and pediatric nephrology. Its format was an interactive PowerPoint presentation (Appendix A) delivered twice virtually and in person by a third-year medical student. No faculty were involved in the delivery of the module, and there was no faculty development necessary. All medical terminology in the module was intended for incoming and current health care professional students. The educational strategies included a didactic section and interactive case discussions. Participants were not required to prepare any content prior to the module, but we recommended that they read an article on race-based clinical algorithms.^8^ Students were instructed to use Poll Everywhere, an online polling application on their electronic devices, during interactive portions of the module. Responses to Poll Everywhere were anonymous, not recorded, and used to promote participant engagement.

We delivered the module to incoming first-year, first-year, second-year, third-year, and fourth-year medical students at the University of Iowa Roy J. and Lucille A. Carver College of Medicine. The same materials were used for all workshops within a 6-month period. There was no limit on the number of participants who could attend the workshops. The workshops were advertised by the Office of Student Affairs and Curriculum, medical school course directors, and student leaders from different associations across the medical school campus. Social media with group membership restricted to Roy J. and Lucille A. Carver College of Medicine students such as GroupMe and a Facebook event page were also used for student recruitment. Three resources were provided in the facilitator guide (Appendix B) to equip facilitators with knowledge of eGFR, race-based clinical algorithms, and basic renal physiology.^8,17,18^

### Session Timeline

This 60-minute module consisted of a preevaluation assessing baseline self-perceived knowledge of race in clinical care and a PowerPoint presentation featuring facilitator introductions, two cases including interactive discussion with students, didactic instruction on the history of the eGFR equation and the race-correction factor, and, finally, key takeaways and time for questions. Students were then invited to complete the postmodule evaluation.

### Data Collection and Analysis

We created pre- and posttest evaluations (Appendix C) in tandem with the module to gauge student reaction and satisfaction at Kirkpatrick level 1.^19^ To ensure anonymity, we did not collect unique identifiers, and pre/post surveys were not linked. The survey questions were aligned with the module learning objectives and scored on 5-point Likert-scales, where lower values indicated less self-perceived knowledge/importance and higher values indicated more self-perceived knowledge/awareness. Additionally, we included two free-response questions in the postevaluation: “What did you like about this workshop?” and “What suggestions do you have to improve this workshop?” We delivered the surveys using Qualtrics, an online data-collection tool, and exported the collected data into Microsoft Excel for analysis. The analysis comprised descriptive statistics and the Mann-Whitney *U* test to assess statistical change between groups before and after the module. Two team members analyzed the narrative data, identifying and summarizing common themes. The project protocol received approval from the Institutional Review Board at the University of Iowa (#202205235).

## Results

We delivered the workshops four times to medical students at various stages of training. Our cohort comprised 55 students in total, with 46 and 55 students completing the pre- and postevaluation, respectively. Table 1 details the breakdown of medical students by their year in training, while Table 2 provides average scores by class level. Mean responses between pre/post surveys are depicted in the Figure. Students exhibited significant growth in self-perceived knowledge of the history of racism in medicine (2.6 vs. 3.2, *p* < .001) and awareness of race in clinical algorithms (2.7 vs. 3.7, *p* < .001), impact of race-based eGFR on quality of life/treatment outcomes (4.5 vs. 4.8, *p* = .01), differences between race and ancestry (3.7 vs. 4.3, *p* < .001), and implications of not removing race from the eGFR equation (2.7 vs. 4.2, *p* < .001). Students showed nonsignificant growth in response to the question of whether the use of race in clinical algorithms was problematic (4.7 vs. 4.8, *p* = .12).

**Table 1.**
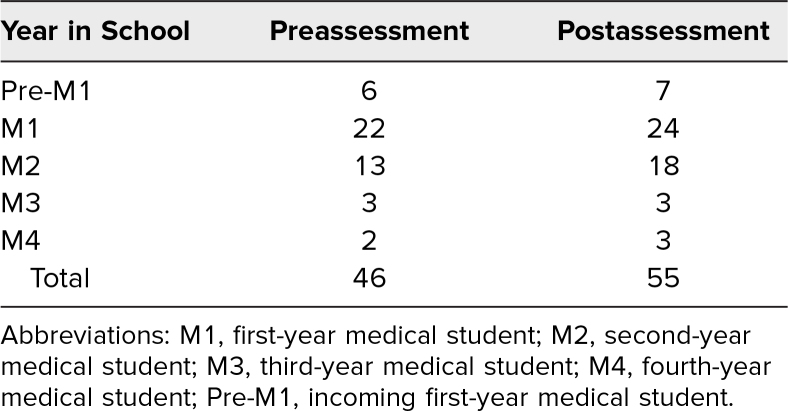
Distribution of Medical Students by Year of Training

**Table 2.**
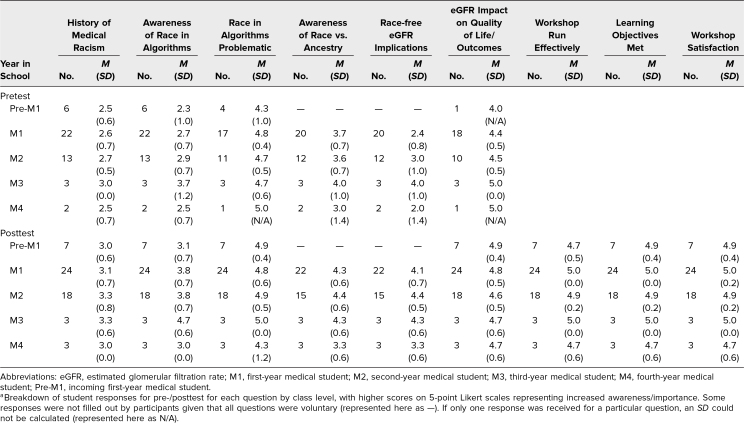
Responses to Pre-/Posttest Questions by Year in Medical School^a^

**Figure.**
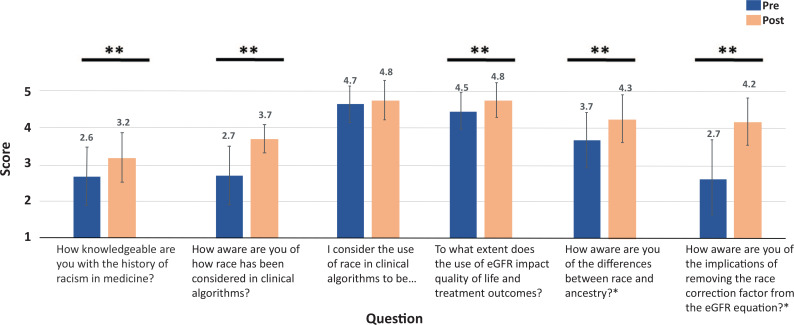
Module pre/post evaluation results: mean scores of students who completed the pretest (*n* = 46) and posttest (*n* = 55). All questions were scored on 5-point Likert-scales where lower values indicated less self-perceived knowledge/importance and higher values indicated more self-perceived knowledge/importance. Standard deviations are presented as error bars. Asterisk (*) indicates a question administered only to current medical students. Double asterisk (**) indicates statistical significance (*p* < .05). Abbreviation: eGFR, estimated glomerular filtration rate.

Additionally, we assessed the efficacy of the workshop by asking attendees, “To what extent do you agree that the workshop objectives were met?” For all four objectives, most students somewhat or strongly agreed that the learning objectives had been achieved. Students shared comments in the free-text portion of the evaluation describing what they liked most about the module. We received a total of 16 responses, with most expressing appreciation for the interactive and informative components of the module, the easy-to-understand format of the presentation, the effectiveness of the facilitator, and the clinical relevance of the cases. Representative comments included the following: “Presenter was open and honest about experiences,” “[I] appreciated the interactive components and open discussion,” “[I] appreciated the great visuals [and] side by side comparisons of the [eGFR calculator],” and “Presentation format [was] well-structured and organized.”

Regarding feedback for enhancing the module, we received a total of 21 responses. Most students indicated a need for additional history about race-correction factors in medicine and how race-based clinical algorithms became prevalent. Other suggestions focused on stylistic improvements, including reducing information density on certain slides and recommending larger graphs and fonts. Some students proposed incorporating more discussion about how race often serves as a proxy measure for socioeconomic status and other biologic variables. Representative comments included the following: “Include additional ways to research this topic,” “More explicit discussion of why race isn't a good proxy,” and “More discussion about how race is a proxy measure of [socioeconomic status], discrimination, racism, etc.”

## Discussion

The misuse of race as a biologic construct in medicine leads to differential treatment of patients and exacerbates inequities in health care.^8^ Without education to appraise the use of race in medical algorithms, health care providers may perpetuate beliefs about biological differences between racial groups and mask underlying drivers of health inequities. The growing need to identify these medical practices makes this module timely for inclusion in medical school and health equity curricula. With several medical institutions eliminating race-based clinical algorithms, the module builds on others’ work to expose the fallacy of race-based algorithms and defines the impact of these changes on health equity. Through interactive case discussions and a didactic presentation, the module was effective in achieving its learning objectives. Students favorably rated the sessions in terms of quality, clarity, and length. Additionally, students had significant growth in their self-perceived awareness and knowledge of (1) the history of racism in medicine, (2) the use of race in clinical algorithms, (3) the differences between race and ancestry, (4) the implications of removing race from the eGFR equation, and (5) the impact of race-based eGFR on quality of life and treatment outcomes.

Based on student feedback, we made several enhancements to the module. Some students requested additional information regarding race correction in medicine and how race serves as a proxy for socioeconomic status and biologic variables. These important topics are beyond the scope of the module. In response, we have created Appendix D, a list of references that can be distributed to students after the module to address in-depth questions and encourage further engagement. Additionally, we have enlarged graphs and font text to enhance clarity. Furthermore, we propose adjusting the language of the question choices on the evaluation forms. We suggest changing the wording from *not at all*, *slightly*, *moderately*, *very*, and *extremely* to *not at all*, *slight*, *moderate*, *aware of*, *very*, and *not applicable/unsure* to ensure that differences in self-perceived knowledge between choices can be reliably captured by the evaluation tool.

Additionally, after our workshops were delivered, we updated the facilitator guide to include new information regarding the race-neutral Chronic Kidney Disease Epidemiology Collaboration equation and kidney transplant availability.^20^ While 60 minutes sufficed for our module, we recommend extending the time to 90 minutes. We propose the additional 30 minutes be allocated to cases 1 and 2 to accommodate their wealth of learning points, foster comprehensive discussion, and heighten the interactivity of the module for learners. We did not conduct a faculty development session as the module was developed and delivered by the same medical student. We suggest incorporating a faculty development session for future facilitators of the module. Finally, to boost student engagement, the module is best delivered in person by facilitators versed in the medical curriculum who can talk about their respective programs’ use of race in medicine.

This module and its evaluation had limitations. Latecomers boosted postquestionnaire engagement without completing the presurvey. We recommend distributing physical QR codes with instructions for both evaluations in all workshops. Despite ambiguous choices in the evaluation form, we observed significant growth across most responses. The absence of individual identification hindered the comparison of self-perceived knowledge before and after the module. Nevertheless, we presented collective awareness using a nonparametric test. The module evaluations were limited, assessing only students’ perceived knowledge of the topic. To comprehensively capture student growth, we recommend including questions about attitudes and content (e.g., social construct of race, unconscious bias in patient care, and social determinants of health). Moreover, free-response questions should be made actionable, such as “Are there things that you would like to change about the workshop?”

Many advanced clinical-year students were unavailable to attend sessions due to variable clinical schedules. We attempted to mitigate this by hosting sessions over the lunch hour, when availability was more open for all students. It is possible clinical students (third- and fourth-years) possessed a higher level of foundational knowledge compared to students in the preclinical phases of medical school (incoming first-years through second-years). As a result, discussions may have diverged based on experience gained during clinical rotations. Unfortunately, small sample sizes in our incoming first-year, third-year, and fourth-year groups prohibited assessing this statistically, but averages by class level are summarized in Table 2. Future research should aim to characterize the effects of the module on students in their clinical phase.

A formal thematic analysis of qualitative data was not possible due to small sample sizes, but we summarized key themes. We aim to gather more qualitative data from students to improve the workshop. Furthermore, because posttest surveys were administered directly after the session, the evaluation represented students’ self-perceived knowledge at one time point. Recognizing that a single session on the topic is unlikely to have long-term efficacy, we recommend integrating this material into the preclinical and clinical curricula. This will benefit students by continually challenging them to reflect on race in medical education throughout their training. Overall, the primary challenge of the module stemmed from its voluntary nature and the lack of comprehensive evaluation. Despite these limitations, we have successfully demonstrated a proof of concept essential for integrating the module into the formal medical school curriculum.

The Liaison Committee on Medical Education, the accrediting body for allopathic medical schools in the US and Canada, does not mandate how race should be addressed in medical education and allows for flexibility in how medical schools should instruct students about health inequity.^21^ Medical schools must make an intentional effort to address how the concept of race is incorporated across all phases of the medical school curriculum. Our module takes the first step in addressing this and complements important dialogue about social determinants of health, diversity in clinical trials, and use of race-correction factors in the published literature. We hope that the module content will also prompt course directors to reevaluate the use of race in medical school lectures. Furthermore, we designed the module with consideration of various training levels, ensuring that students at all stages of medical school can comprehend the content. This design allows schools the flexibility to seamlessly integrate the module into both preclinical and clinical curricula. While originally intended for medical students, the module is useful for students in other health disciplines (nursing, physician assistant, pharmacy, etc.) who play a critical role in patient care. Moving forward, we hope to integrate the module into existing medical school curricula. Overall, by exposing the fallacy of race-based algorithms and its impact on health equity, we hope to help medical trainees build a foundation to identify and appraise the appropriate use of race in medical practice throughout their training.

## Appendices


Presentation.pptxFacilitator Guide.docxEvaluation Forms.docxResources for Interested Students.docx
*All appendices are peer reviewed as integral parts of the Original Publication.*
