# Supplementary material for: Reassessing the Use of Race in Clinical Algorithms: An Interactive, Case-Based Session for Medical Students Using eGFR
Source: MedEdPORTAL. 2024 Jun 21;20:11412. doi: 10.15766/mep_2374-8265.11412 (PMC11219082; doi:10.15766/mep_2374-8265.11412)
Supplement: Supplementary file 1 — Presentation.pptxFacilitator Guide.docxEvaluation Forms.docxResources for Interested Students.docx [file mep_2374-8265.11412-s001.zip › B. Facilitator Guide.docx]

**Appendix B: Facilitator Guide**

**Directions:** Welcome to the facilitator preparation document for “Reassessing the Use of Race in Clinical Algorithms: An Interactive Case-Based Session for Medical Students Using eGFR.” Please take a few moments to look over the session objectives and suggested peer-reviewed articles below. You can also find the suggested agenda with a timeline. There is an option to complete this module including evaluations in 60 minutes but we suggest allocating a total of 90 minutes, with the additional 10 and 20 minutes allocated for Case 1 and Case 2, respectively (see timeline on Page 4).

**Overview:**

**Session Objectives:**

- - - 1. Discuss how race is used in clinical algorithms, emphasizing the eGFR equation.
      2. Explain how race-based clinical algorithms can magnify existing inequalities in healthcare.
      3. Identify differences between race, ancestry, and genetics.
      4. Describe the implications of removing the race correction factor from the eGFR equation.

**Audience:** Healthcare professional students (i.e., medical students, nursing students, etc.)

**Suggested Requirements and Qualifications for Facilitator:**

- This session needs one facilitator (medical student, faculty, or other healthcare professionals) who is versed in medical education topics and has a basic understanding of renal physiology.
- We recommend spending at least 3-4 hours reviewing the material in this facilitator preparation document and the speaker notes listed in the PowerPoint prior to presenting the module.
- We suggest that the facilitator read the following resources to become familiarized with the current use of race in clinical algorithms and its implications.
  - A primer on race-based clinical algorithms: Vyas DA, Eisenstein LG, Jones DS. Hidden in Plain Sight - Reconsidering the Use of Race Correction in Clinical Algorithms. N Engl J Med. Aug 27 2020;383(9):874-882. doi:10.1056/NEJMms2004740
  - The relationship between serum creatinine and eGFR: Gounden V, Bhatt H, Jialal I. Renal function tests. 2018.

- - The eGFR equation, the race-based correction factor, and implications of removing the correction factor: Tsai JW, Cerdeña JP, Goedel WC, et al. Evaluating the Impact and Rationale of Race-Specific Estimations of Kidney Function: Estimations from U.S. NHANES, 2015-2018. *EClinicalMedicine*. Dec 2021;42:101197. doi:10.1016/j.eclinm.2021.101197

**A/V Needs:** Screen, Individual Device with Internet Access (Per Participant)

**PowerPoint**: Yes – Appendix A

**Table of Contents:**

Slide Instructions: 4

Slide 1: Reassessing the Use of Race in Clinical Algorithms:

An Interactive Case-Based Session for Medical Students Using eGFR 5

Slide 2: Disclosures 5

Slide 3: Getting Started: Patient Case 5

Slide 4: Patient Case 5-6

Slide 5: Q1: Do you think clinicians integrate race into their clinical decision-making? _______7

Slide 6: Q1 Continued PollEv Question 7

Slide 7: Learning Objectives 7

Slide 8: Case #2: Patient Encounter 7

Slide 9: Patient Encounter cont. 7-8

Slide 10: Family History and Social History 8

Slide 11: Review of Systems and Physical Exam 8-9

Slide 12: Labs 9

Slide 13: Problem list 9-10

Slide 14: What can we do for this patient? 10-11

Slide 15: What is eGFR? 11

Slide 16: What is eGFR cont.? 11

Slide 17: How bad is her kidney function now? 11-12

Slide 18: Creation of the eGFR equations 12

Slide 19: Creatinine and Race, what’s the connection? 13

Slide 20: Differences between races in the CKD-EPI equation 13

Slide 21: CKD-EPI equation: a timeline 13-14

Slide 22: Race versus Ethnicity - Important Definitions 14-15

Slide 23: …. Versus Ancestry and genetics - important distinctions to make 15

Slide 24: Implications of dropping the race correction Factor 15-16

Slide 25: Back to the case! 16

Slide 26: How have other institutions handled this dilemma? 16

Slide 27: What other specialties have race-based algorithms? 16

Slide 28: Takeaways 16

Slide 29: References 16

Slide 30: Acknowledgments_____________________________________________________17

Slide 31: Questions_____________________________________________________________17

#

# **Slide Instructions:**

Prior to and directly after delivering the workshop, students should fill out the pre and post evaluation form (Appendix C), respectively, to assess their baseline perceived knowledge. We recommend printing out QR codes with a link to the evaluation forms that are readily available for students. You may also consider having students read Vyas et al. (2020)^1^ prior to participating in the workshop. This is highly encouraged but not mandatory for student participation.

1. Vyas DA, Eisenstein LG, Jones DS. Hidden in Plain Sight - Reconsidering the Use of Race Correction in Clinical Algorithms. N Engl J Med. Aug 27 2020;383(9):874-882. doi:10.1056/NEJMms2004740

This workshop may be completed in approximately 60 minutes (red text). To allow for more discussion, we’ve included additional time in Case 1 and Case 2 (blue text) for a total session time of 90 minutes.

**Suggested Agenda and Timeline:**

- Pre-module survey (3 minutes)
- Introduction/Case #1 Discussion: Slides 1-7 (10 minutes or 20 minutes)
- Case #2 Discussion: Slides 8-17 (10 minutes or 30 minutes)
- eGFR History: Slides 18-21 (14 minutes)
- Race, Ancestry, and Genetics Discussion, and Dropping Correction Factor: Slides 22-24 (12 minutes)
- Back to the Case, Other Institutions, Other Specialties: Slides 24-27 (6 minutes)
- Takeaways: Slide 28 (2 minutes)
- Questions: Slide 31 (3 minutes)
- Post-Module Evaluation (3 minutes)

**NOTE:** Suggested scripts and a facilitator guide can be found in the speaker notes located in the PowerPoint (Appendix A). Of note, any content in *italicized font* are instructions for the facilitator and are not meant to be read out loud to the students.

**Slide 1:** Reassessing the Use of Race in Clinical Algorithms: An Interactive Case-Based Session for Medical Students Using eGFR

- *Add the names, credentials, and background information of the facilitator to the slide.*
- *The facilitator should introduce themselves to the audience and discuss their roles at their institutions.*
- *Facilitators may mention that they are following the content and guidelines of this peer‐reviewed module.*
- *Students should enter the PollEV link (QR code) in their personal devices prior to participating in this module and complete the pre-module assessment. Remind students that responses will be kept anonymous. Consider the following script:*

This presentation is designed to be interactive. We will be going over two short cases to guide today’s talk on the fallacy of using race in clinical algorithms by focusing on race correction factor in the eGFR equation. Briefly, we will cover the important differences between race, ancestry and genetics, and end with what you can do to advocate against race-based clinical algorithms. Before we begin, please make sure you enter the PollEv link (QR code) into your mobile devices. Your submitted responses will be kept anonymous.

# **Slide 2:** Disclosures

- *If present, make sure to include any financial or other conflicts of interest relevant to the talk. If none are present, you may include: “I have no financial or other conflicts of interest relevant to this talk.”*

# **Slide 3:** Getting started: Patient Case

- *You may choose to approach this in one of three ways: (1) make a student read off the case word-for-word on the slide to be more interactive, (2) summarize pertinent positives and negatives of the case, or (3) have the facilitator read directly off the slide. Either way, make sure to highlight the following two key points:*
  - *The patient was denied pain medication when she was indeed in pain.*
  - *The patient had to provide concrete evidence of disease (i.e., with a CT scan) to get pain medication.*

# **Slide 4:** Patient Case

- *Click through the animations illustrating Dr. Susan G. Moore’s story and pose the two questions included in the script below to the audience. Allow ~3-4 minutes for a response and discussion session.*
- *As you go forward to Case 2, it is important to emphasize that while scientific racism and the use of race as a biological construct is intimately linked to physician implicit/explicit racism, and racist policies/practices in health care, they are separate phenomena.*

*Consider the following script:*
This case illustrates the final days of a real-life doctor by the name of Dr. Susan G. Moore. Dr. Moore was a geriatric-family medicine physician in Grand Rapids, MI. Her story went viral when she posted her hospital course on Facebook Live, where hundreds of people would tune in to get live updates regarding her care. Given this patient's case, we have two questions for you to reflect on and discuss. These two questions highlight our key themes for the rest of the talk:

What does Dr. Moore’s story illustrate?

- 1. It illustrates how healthcare providers may not always listen to their patients, and there may be an implicit bias arising from a racial component. For example, patients with sickle cell anemia are frequently ignored, and black people who express concerns about pain are often undertreated/minimized in healthcare^3^.

Did her race play a factor in the medical care she did (not) receive? If so, how?

- 1. You may or may not agree that her race played a factor in her medical care as unconscious biases on behalf of providers are often difficult to recognize. Dr. Moore had to prove that her pain had a physical explanation (i.e., with a CT scan) when we know that pain is as much psychologic as it is physical.

Overall, Dr. Moore’s story illustrates some of the racial injustices that Black people face when accessing healthcare. The poor treatment that Dr. Moore faced poses the following question: “How would Black people who do not have medical training be able to advocate for themselves in these situations?” These issues are being highlighted by several prominent celebrities who have publicly spoken out about their experiences and those of people of color in the healthcare system. Current vice-president Kamala Harris and singer Beyonce are two prominent black folx who have publicly expressed concerns regarding the unacceptably high maternal mortality rates among black mothers. Others such as accomplished tennis player Serena Williams and sprinter Alyson Felix have also spoken out about such issues.^3^ Kareem Abdul Jabbar and Bernie Mac also advocate for equity in healthcare for Black Americans.

1. Hoffman KM, Trawalter S, Axt JR, Oliver MN. Racial bias in pain assessment and treatment recommendations, and false beliefs about biological differences between blacks and whites. *Proc Natl Acad Sci U S A*. Apr 19 2016;113(16):4296-301. doi:10.1073/pnas.1516047113
2. Global, regional, and national levels of maternal mortality, 1990-2015: a systematic analysis for the Global Burden of Disease Study 2015. *Lancet*. Oct 8 2016;388(10053):1775-1812. doi:10.1016/s0140-6736(16)31470-2

# **Slide 5:** Question 1: Do you think clinicians integrate race into their clinical decision-making?

- *Read the question provided on the slide to the students.*
- *Ask them to submit their responses using the PollEv (QR code) application on their mobile devices. Provide them with approximately ~1 minute to respond.*
- *For our talk, we used the “Q/A” activity type. Make sure the students are not able to see the responses from their classmates.*

# **Slide 6:** Question 1 Continued PollEV Question

- *Unmute the responses and show the most voted answer to the group.*
- *Explain that this question is intended for students to reflect on their responses.*
- *Regardless of the results, the goal is that by the end of this lecture, students will be able to answer this question definitively.*

# **Slide 7:** Learning Objectives

- *Introduce the learning objectives to the students.*
- *Explain that students should be able to complete each learning objective by the end of this session.*

# **Slide 8:** Case #2: Patient Encounter

- *Note that you will introduce the second case of the presentation.*
- *For students that are not yet in the clinical portion of their curriculum, reassure them that this part of the presentation does require too much clinical background, and it is intended only for discussion. Consider the following script:*

We will now go through the second case. For those students who are still in the pre-clinical curriculum, some of this content will be new in the way that it is presented. You will only be expected to pick up big themes from this case so please do not try to fixate on individual lab values or specific physical exam findings. I will also be explaining key takeaways as we go through the case.

# **Slide 9:** Patient Encounter Continued

- *Read the first two lines of the patient encounter to the students. You may choose to summarize the past medical history and medication list, or you may read the content from the slide. Consider the following script:*

AT is a 43 y/o Black woman with an 8-year history of uncontrolled Type 2 Diabetes and Hypertension presenting to urgent care with a chief complaint of left foot pain.

On the left side of this slide is AT’s past medical history. There are several indications that point to metabolic syndrome including obesity, type 2 diabetes, hyperlipidemia, and hypertension. AT also has Stage IV chronic kidney disease secondary to uncontrolled diabetes and has hypothyroidism. Her medications are listed on the right side of the slide. She has several blood pressure medications (lisinopril and amlodipine), hypoglycemic agents for diabetes (metformin), a statin for hyperlipidemia, and Gabapentin/Naproxen for neuropathic pain. Hypothyroidism is being managed with levothyroxine. She has also been given a course of trimethoprim/sulfamethoxazole for an infection of a diabetic foot ulcer.

# **Slide 10:** Family History and Social History

- *You may choose to summarize the family history and social history, or you may read from the slide. Consider the following script:*

*Family History:*

Her family history is given below. Both of her parents are deceased. Mom died of a myocardial infarction at 65 and father died from a stroke at 43 years. She has one brother who is legally blind and has type 2 diabetes, and two sisters. One of these sisters has type 2 diabetes, obesity, and hypothyroidism. The other sister has chronic back pain treated with opioids.

*Social History:*

She is married with four sons whose ages are listed. She works full-time at a desk job for 50 hours/per week and gets limited exercise. Notably, she has been out of her medications for the past week.

# **Slide 11:** Review of Systems and Physical Exam

- *Depending on your audience (preclinical versus clinical students), you may choose to summarize the review of systems (ROS) and physical exam to mention pertinent positives, or you may read off the slide.*
- *Pertinent positives in the physical exam section are bolded. Make sure to clarify any medical jargon that is unclear to students (i.e., what is a review of systems, what is dysgeusia, what does serosanguinous mean, etc.) Consider the following script:*

*Review of systems:*

Her review of systems is as follows: she is positive for severe left foot pain, nausea, itching, claudication, snoring, fatigue, and dysgeusia. Dysgeusia is a metallic taste in the mouth indicative of electrolyte abnormalities, among many other causes.

*Physical Exam:*

Her physical exam is given here. Her vital signs are significant for elevated blood pressure, and low oxygen saturation at 91% on room air. Physical exam was significant for labored breathing, stasis dermatitis over shins bilaterally, and a foot ulcer over the left plantar metatarsal that is draining serosanguineous fluid. The respiratory exam was significant for faint bibasilar crackles over the lower lung lobes. The cardiovascular exam demonstrated a Grade 2 holosystolic murmur auscultated over the apex that radiates to the axilla with +2 pitting edema over shins bilaterally. Neuro exam was significant for decreased sensation over both extremities up to the shin region.

# **Slide 12:** Labs

- *You may choose to summarize the labs. We recommend not spending too much time on individual lab values. The main takeaway from this slide is to show that the patient has deteriorating kidney function. This is clear from the given electrolyte abnormalities and elevated creatinine. If summarizing, make sure to include the following key points:*
- *The patient has severe electrolyte abnormalities. This elevated serum creatinine and BUN are suggestive of poorly functioning kidneys.*
- *The patient has an elevated white blood cell count, likely indicative of infection.*
- *The patient has poorly controlled serum lipids, hypothyroidism, and diabetes (as evidenced by HbA1c above goal). Consider the following script:*

The patient's labs are provided with corresponding reference ranges. The basic metabolic panel is significant for several electrolyte abnormalities including hyperkalemia and metabolic acidosis. These labs along with increased blood urea nitrogen (BUN) and serum creatinine suggest that her kidneys are poorly functioning. Her complete blood count (CBC) shows elevated white blood cells and low hemoglobin, indicative of some sort of infection or systemic physiological stressor. Her lipid panel and endocrine labs indicate that she has poorly controlled hyperlipidemia, hypothyroidism, and type 2 diabetes.

# **Slide 13:** Problem list

- *Ask the group what else should be on their problem list.*
- *A problem list is what clinicians use to organize their thoughts when caring for patients. It lists all the problems that the patient has and can include strictly medical items (e.g., worsening kidney function, infection, diabetes) to biopsychosocial factors that need to be considered as part of the patient’s care.*
- *Before clicking through the suggested responses on the slide, make sure to prompt the students about their problem list. Encourage them to think broadly. Consider the following script:*

A problem list is used to identify the patient’s current problems that need to be addressed. What problems can you all think of that we need to address as a clinician in both acute and chronic settings?

# **Slide 14:** What can we do for this patient?

- *Guide the students through the questions on the slides.*
- *Reveal the answers only after the students have had a chance to answer them. Consider the following script:*

Pain: what medications are available to us?

One of the first things that the patient is concerned about is pain. This is at the top of our problem list. What medications are available to us to treat pain? *Allow students to discuss.*

*Reveal answer*: non-Steroidal anti-inflammatory medications (Ibuprofen, Naproxen), acetaminophen, opioids, gabapentin/pregabalin, tricyclic antidepressants, duloxetine. What do we prefer here?

- *Response: We will most likely avoid NSAIDs as they can be nephrotoxic, especially if this patient has a pre-existing renal insufficiency. Opioids are generally not good options for neuropathic pain, but they should be considered. Gabapentin, TCAs, and duloxetine may be good options for long-term neuropathic pain control but will need to be dose-titrated given her poor kidney function.*

Given what we know of her racial and social background, how will we choose to manage her pain?

- *Response: May ask follow-up questions depending on what students say. A reasonable question could be, “Does the fact that her sister uses opioids for her back pain affect our decision to prescribe opioids for this patient?” There are no right or wrong answers. The main idea is to explore the different options with students and probe for any hidden biases.*

Based on her social situation, what support do you feel she needs to return for a follow-up?

*Reveal suggestions (not exhaustive):*

- Mental Health: Is there possibly an element of anxiety/depression that we are missing?
- Should we involve social work or physical therapy?
- Insurance issues?
- Other structural barriers that are preventing care (e.g., lack of transportation, lack of social support, etc.)

Lastly, regarding the patient’s kidney function, what is it? How do we measure it?

*This question is meant to be a transition point to the next slide and not a question that students need to answer. For advanced students with clinical experience, this may be a good question to ask them.*

# **Slide 15:** What is eGFR?

- *Read all the content on the slide.*
- *Make sure to explain the relationship between serum creatinine and eGFR. The figure may be used to illustrate the relationship between serum creatinine and eGFR. The black line in the figure illustrates normal serum creatinine in a healthy, normal weight adult.*
- *Students should understand that this is the best metric to assess kidney function.*
- *Highlight the limitations of using creatinine to estimate eGFR in select populations.*
- *Consider the following script:*

eGFR is a calculation that estimates kidney function based on how well our kidneys filter biological agents such as creatinine and cystatin C. eGFR, as demonstrated in the figure, is inversely and non-linearly related to serum creatinine. The black line illustrates a normal serum creatinine (0.7-1.3 mg/dL) in a healthy, normal weight adult. We can see that even small changes in serum creatinine below/above the normal range can change eGFR dramatically. As illustrated, the association between serum creatinine and eGFR is not perfect. There are other factors that are considered in the eGFR equation such as age and sex to make the calculations more accurate. These variables are important in calculating the eGFR, and are used by MDRD, CKD-EPI equations.

# **Slide 16:** What is eGFR cont.?

- *Consider the following script:*

While the eGFR is a simple number to calculate, this value has major implications for clinical care. It can be used as a benchmark for kidney transplant referral, the dosage of medications, and assessment of nephrotoxicity (e.g. warfarin, Lithium, NSAIDS, etc.)

# **Slide 17:** How bad is her kidney function now?

- *Read all the content on the slide.*
- *Mention all the variables used to calculate eGFR.*
- *In this case, AT’s variables are age, serum creatinine, and race.*
- *Use this value and compare the calculated eGFR value to where AT would fall in the CKD staging classification.*
- *AT would fall under Stage IV CKD, but she is not eligible for a transplant (important takeaway point). However, she is still a candidate for a nephrology referral.*
- *The literature has noted that patients are often denied transplant referrals because their kidney function was not deemed “bad enough.” The following resource may be provided to students if they are interested in knowing how the transplant referral process has been impacted by eGFR.^4^*

1. Ahmed S, Nutt CT, Eneanya ND, et al. Examining the Potential Impact of Race Multiplier Utilization in Estimated Glomerular Filtration Rate Calculation on African-American Care Outcomes. *J Gen Intern Med*. Feb 2021;36(2):464-471. doi:10.1007/s11606-020-06280-5

# **Slide 18:** Creation of the eGFR equation

- *Make sure to emphasize the relationship between eGFR and creatinine.*
- *Important to note that explaining the details of the Levey study can be confusing for a lot of students. We suggest dedicating time to the content of this slide. Consider the following script:*

One of the first studies to quantify the supposed biological differences between black and white people was done on children. Harsha et al. showed that black kids had less body fat and therefore higher muscle mass than their white counterparts. This was one of the pieces of evidence cited in the Levey et al. paper and was instrumental in forming the original MDRD equation (eGFR estimation).

This study used ^125^I-iothalamate, a chemical that can be injected into the body and measured in the urine. It is not secreted or reabsorbed by the tubules of the kidney. Therefore, it is considered the gold standard for measuring kidney function. Researchers took 1628 patients with CKD and measured their serum creatinine and their kidney function using this gold standard test. From there, they derived equations to back-calculate which variables could be put into a model to best estimate the gold-standard equation. The results of this regression analysis are plotted in the figure. They identified several independent factors associated with lower eGFR such as higher serum creatinine concentration, older age, female sex, non-black ethnicity, higher serum urea nitrogen levels, and lower serum albumin.

An equation was formulated to best estimate kidney function. However, a racial correction factor was added, making eGFR 18% higher for black patients. Notice in the figure that at the same creatinine value *(y-axis in the figure*), black people have higher eGFRs than their white counterparts. This is most evident at the lower eGFR ranges. Therefore, the idea was that this discrepancy must be corrected. This was further supported by the authors from Harsha’s study done 20 years prior which showed that black children had greater muscle mass than white children. The reasoning was that if black people had higher muscle mass and were clearing more creatinine, this needed to be accounted for. Nevertheless, there were several critical problems with the study’s design. Among many, 80% of the study population was white and only 12% was black. Furthermore, it was not defined what “black” meant in the study. Was this self-identification or was someone assigning this identity? What about children that are of mixed race?

# **Slide 19:** Creatinine and Race, what’s the connection?

- *Read all the content on the slide. Consider the following script:*

According to Levey’s paper, there must be a biological reason for the difference in creatinine and eGFR between black and white people. Hsu et al (2008) set out to elucidate this relationship. They showed that the prevalence of elevated serum creatinine was significantly higher in black populations versus non-black populations. Notably, this was maintained even after adjusting for muscle mass. Furthermore, these differences were not readily explained by nutritional status or body composition. This suggests that there had to be other factors influencing creatinine levels other than muscle mass. Given the many problems with the MDRD equation, a new equation was needed to estimate kidney function which led to the development of the CKD-EPI equation.

# **Slide 20:** Differences between races in the CKD-EPI equation

- *Explain that on the left of the slide is AT’s kidney function and on the right of the slide is her non-black equivalent. By looking at these values, we can conclude that if these values were used as a basis for a transplant referral, AT would not qualify.*

# **Slide 21:** CKD-EPI equation: a timeline

- *Illustrated on this slide is a timeline of events from the conception of the MDRD equation to the present day.*
- *Click through the animations while explaining these events.*
- *In 2012, the CKD-EPI equation was refitted with cystatin C, a molecule filtered by kidneys that seemingly addressed the shortcomings of creatinine. Moreover, an equation with creatinine and cystatin C demonstrated higher accuracy than either equation used alone.*
- *The three thunderbolts represent the social unrest that happened in 2020. This period forced different institutions to reconsider how they were addressing race, including the healthcare system.*
  - *It’s important to note that BLM was not the first time there was dissenting medical opinion in the medical literature regarding race and medicine/health (e.g. works of W.E.B. du Bois and Kelly Miller that challenged spirometry race-correction factors^5^, work of the Black Panther group that exposed medical bias of patients with Sickle Cell Disease^6^), however, BLM was a movement that catalyzed concrete change in institutional policies*
- *Highlight the important role that medical students had in this movement. Medical students at the University of Washington were the first to advocate for removing the race correction factor. This catalyzed change within the institution and helped modify national guidelines.*
- *Given the evolving nature of the eGFR race correction factor debate, we encourage facilitators to research current events relating to use of the race-neutral CKD-EPI equation such as: changing transplant referral practices, nephrology referrals, etc. For example, by the time this facilitators guide was created, the US Organ Procurement and Transplantation Network [OPTN] began requiring transplant centers to modify transplant list wait times for black patients using the race-neutral CKD-EPI equation.^7^*

5. Lujan HL, DiCarlo SE. Science reflects history as society influences science: brief history of "race," "race correction," and the spirometer. Adv Physiol Educ. Jun 1 2018;42(2):163-165. doi:10.1152/advan.00196.2017

6. Bassett MT. Beyond Berets: The Black Panthers as Health Activists. Am J Public Health. Oct 2016;106(10):1741-3. doi:10.2105/ajph.2016.303412

7. Mohottige D, Purnell TS, Boulware LE. Redressing the Harms of Race-Based Kidney Function Estimation. *Jama*. Mar 21 2023;329(11):881-882. doi:10.1001/jama.2023.2154

#

# **Slide 22:** Race versus Ethnicity - Important Definitions

- *This slide details distinctions between race and ethnicity. At this time, you may have students volunteer definitions or use a think-pair-share method. If student proposed definitions allude to a biologic basis for race, be sure to comment on it. Example of a response to a student could be,* “*This represents a widely accepted notion of race and is frequently employed in modern medical research – we will spend time talking about this today.”*
- *Read off the definitions and make sure to highlight the key differences between the underlined terms as you click through the slide – race is often hierarchical, meaning it has been used to categorize human populations into distinct groups with perceived differences in abilities, characteristics, or value.*
  - *If you have time, consider also offering epidemiologist, Dr. CP Jones’, definition of race^8^ “race is a social classification in our race-conscious society that conditions most aspects of our daily life experiences and results in profound differences in life chances”*
- *Race is frequently used in medicine to describe black as a “risk factor” (i.e., a risk factor for poorly controlled hypertension, increased maternal morbidity/mortality, etc.).*
- *This rhetoric is harmful as it can perpetuate the idea that being Black is inherently associated with the disease. Rather, we need to focus on the context behind these diseases to explain these associations (i.e., lack of access to healthcare, intergenerational trauma, etc.). Another way this point can be stated is that racism is the driver of inequities, rather than race alone.*
- *The questions in the box are for students to reflect on their own. These questions are not meant to be discussion points but are meant to make students think. If students would like to discuss these points, then we suggest that they should be given an opportunity to do so during the workshop.*
- *You may also discuss the existence of mixed-race individuals and how this puts the onus on the clinician to assign a race when using these equations. This further illustrates how these equations become less rigorous in practice and exposes the subjectivity of race-correction factors.*

8. Jones CP. Invited commentary:“race,” racism, and the practice of epidemiology. *American journal of epidemiology*. 2001;154(4):299-304.

# **Slide 23:** …. Versus Ancestry and genetics - important distinctions to make

- *This slide expands on the last slide and details important distinctions between ancestry and genetics. Read off the definition by Yudell et al., 2016 on ancestry and make sure to highlight the key differences between ancestry and genetics as you click through the slide*
  - *Key difference to highlight is that ancestry is about genealogy (researching and documenting relationships, lineage, and the history of individuals and families over time) versus genetics which is about the empiric study of genes and transmission of traits from one generation via genetic code*
- *If the term “Black” is used, it should be made clear whether we are talking about race, ancestry, or whether there is a genetic basis for the disease association or another reason that has not been identified.*
- *Key takeaway from this discussion is that Race ≠ ancestry or genetics.*

# **Slide 24:** Implications of dropping the race correction factor

- *There are several implications in patient care when dropping the race correction factor.*
- *Read all the content on the slide.*
- *Help students understand that there is a balance between under and over-diagnosing kidney disease (goldilocks phenomenon). For example, after dropping the race correction factor, more people will be diagnosed with more advanced kidney disease. This increase may have implications for the burden of disease on the healthcare system.*
- *The inclusion of Cystatin C may be a viable option moving forward. However, Cystatin C is not covered by all types of insurance and the Veterans Affairs system does not have it as part of its standard lab set. Therefore, this will have to be a send-out lab for a routine lab measurement. This also makes Cystatin C difficult to adopt in rural settings.*
- *Pose the question in the box to students and ask them for their thoughts after explaining the pros and cons of removing the race correction factor.*

# **Slide 25:** Back to the case!

- *This slide includes AT’s eGFR as previously shown. However, it also shows the most up-to-date non-race-based CKD-EPI 2021 equation.*
- *Emphasize that this case is about health equity.*
- *As illustrated, AT would be eligible for a kidney transplant based on the latest equation. Again, be sure to not over-simplify the transplant process to just the eGFR value as stated before in Slide 16.*

# **Slide 26:** How have other institutions handled this dilemma?

- *Read the latest guidelines by the National Kidney Foundation and American Society of Nephrology Joint Task Force provided on the slide.*
- *The box provided is intended for you to include what your institution currently practices regarding the eGFR equation (race or non-race-based).*
- *The institutions at the right side of the slide are some of the first institutions to drop the race-based correction factor in the eGFR equation.*

# **Slide 27:** What other specialties have race-based algorithms?

- *This slide displays a non-exhaustive list of medical specialties still using race correction factors. This slide is meant to illustrate the ubiquitous use of race correction factors across medicine.*
- *You do not need to read through every specialty but do highlight some of them. This is an example of race-based medicine.*

# **Slide 28:** Takeaways

- *Share the three takeaways on the slide that all students should remember after completing the workshop.*
- *Mention that medical students can critically appraise the primary literature and have the right to question practices regarding race-based medical care.*

# **Slide 29:** References

# **Slide 30:** Acknowledgments

- *May choose to mention the original creators of this presentation here (authors of the manuscript).*

# **Slide 31:** Questions

- *Allow participants to ask any last-minute questions.*
- *Present the post-module survey after answering questions from the audience.*
- *We encourage facilitators to print out the QR code so that participants can scan it if their device does not scan from a distance.*
