# Supplementary material for: Reassessing the Use of Race in Clinical Algorithms: An Interactive, Case-Based Session for Medical Students Using eGFR
Source: MedEdPORTAL. 2024 Jun 21;20:11412. doi: 10.15766/mep_2374-8265.11412 (PMC11219082; doi:10.15766/mep_2374-8265.11412)
Supplement: Supplementary file 1 — Presentation.pptxFacilitator Guide.docxEvaluation Forms.docxResources for Interested Students.docx [file mep_2374-8265.11412-s001.zip › C. Evaluation Forms.docx]

**Directions:** Deliver these pre and post-assessment forms to the students directly prior to and after facilitating the presentation. Pre-assessment and post-assessment forms are designed to assess the student’s change in self-perceived knowledge of race correction factors before and after the educational intervention respectively.

**Pre-Assessment**

1. Age: __________________
2. Year in medical school: ___
3. How knowledgeable are you with the history of racism in medicine?
4. Not At All knowledgeable
5. Slightly Knowledgeable
6. Moderately Knowledgeable
7. Very Knowledgeable
8. Extremely Knowledgeable
9. How aware are you of how race has been considered in clinical algorithms?
10. Not At All Aware
11. Slightly Aware
12. Moderately Aware
13. Very Aware
14. Extremely Aware
15. I consider the use of race in clinical algorithms:
16. I don’t know
17. Not at all a problem
18. Minor Problem
19. Moderate Problem
20. Serious Problem
21. How aware are you of the differences between race and ancestry?
22. Not At All Aware
23. Slightly Aware
24. Moderately Aware
25. Very Aware
26. Extremely Aware
27. How aware are you of the implications of removing the race correction factor from the eGFR equation?
28. Not At All Aware
29. Slightly Aware
30. Moderately Aware
31. Very Aware
32. Extremely Aware
33. To what extent does the use of eGFR impact quality of life and treatment outcomes.
34. I don’t know
35. No Impact
36. Minor Impact
37. Moderate Impact
38. Extreme Impact

**Post-Assessment**

1. Age: __________________
2. Year in medical school: ___
3. How knowledgeable are you with the history of racism in medicine?
4. Not At All knowledgeable
5. Slightly Knowledgeable
6. Moderately Knowledgeable
7. Very Knowledgeable
8. Extremely Knowledgeable
9. How aware are you of how race has been considered in clinical algorithms?
10. Not At All Aware
11. Slightly Aware
12. Moderately Aware
13. Very Aware
14. Extremely Aware
15. I consider the use of race in clinical algorithms:
16. I don’t know
17. Not at all a problem
18. Minor Problem
19. Moderate Problem
20. Serious Problem
21. How aware are you of the differences between race and ancestry?
22. Not At All Aware
23. Slightly Aware
24. Moderately Aware
25. Very Aware
26. Extremely Aware
27. How aware are you of the implications of removing the race correction factor from the eGFR equation?
28. Not At All Aware
29. Slightly Aware
30. Moderately Aware
31. Very Aware
32. Extremely Aware
33. To what extent does the use of eGFR impact various spheres of a person’s life.
34. I don’t know
35. No Impact
36. Minor Impact
37. Moderate Impact
38. Extreme Impact

**Satisfaction –** Please rate your satisfaction with the overall workshop.

1. The workshop was run effectively.
2. Strongly Disagree
3. Somewhat Disagree
4. Neither Agree nor Disagree
5. Somewhat Agree
6. Strongly Agree
7. The workshop learning objectives were met.
8. Strongly Disagree
9. Somewhat Disagree
10. Neither Agree nor Disagree
11. Somewhat Agree
12. Strongly Agree
13. I was satisfied with this workshop.
14. Very Dissatisfied
15. Somewhat dissatisfied
16. Neither Satisfied nor Dissatisfied
17. Somewhat Satisfied
18. Very Satisfied

What did you like about this workshop? Please elaborate.

___________________________________________________________________________

___________________________________________________________________________

What suggestions do you have to improve this workshop?

___________________________________________________________________________

___________________________________________________________________________
