# Supplementary material for: Reassessing the Use of Race in Clinical Algorithms: An Interactive, Case-Based Session for Medical Students Using eGFR
Source: MedEdPORTAL. 2024 Jun 21;20:11412. doi: 10.15766/mep_2374-8265.11412 (PMC11219082; doi:10.15766/mep_2374-8265.11412)
Supplement: Supplementary file 1 — Presentation.pptxFacilitator Guide.docxEvaluation Forms.docxResources for Interested Students.docx [file mep_2374-8265.11412-s001.zip › D. Resources for Interested Students.docx]

**Appendix D.** **Resources for interested students**

**Directions**: The following references are for students who are interested in further researching the topics of eGFR, race-based correction factors, and more

**1. Race-based correction factors:**

- Vyas DA, Eisenstein LG, Jones DS. Hidden in Plain Sight - Reconsidering the Use of Race Correction in Clinical Algorithms. *N Engl J Med*. 2020 Aug 27;383(9):874-882. doi: 10.1056/NEJMms2004740.
- Tong M, Artiga S. Use of race in clinical diagnosis and decision making: overview and implications. *KFF*. 2021 Dec 9.
- Braun, L., Wentz, A., Baker, R., Richardson, E., & Tsai, J. (2021). Racialized algorithms for kidney function: Erasing social experience. *Social Science & Medicine*, *268*, 113548. https://doi.org/10.1016/j.socscimed.2020.113548

**2. Origins of the MDRD equation:**

***Original study***

- Levey AS, Bosch JP, Lewis JB, *et al*. A more accurate method to estimate glomerular filtration rate from serum creatinine: a new prediction equation. Modification of Diet in Renal Disease Study Group. *Ann Intern Med*. 1999 Mar 16;130(6):461-70. doi: 10.7326/0003-4819-130-6-199903160-00002.

***Research cited by Levey et al. regarding the association between higher muscle mass and black people***

- Cohn SH, Abesamis C, Zanzi I, *et al*. Body elemental composition: comparison between black and white adults. American Journal of Physiology-Endocrinology And Metabolism. 1977 Apr 1;232(4):E419.
- Harsha DW, Frerichs RR, Berenson GS. Densitometry and anthropometry of black and white children. *Human biology*. 1978 Sep 1:261-80.

**3. National Kidney Foundation and American Society of Nephrology Joint Task Force Recommendations:**

- Delgado C, Baweja M, Crews DC, *et al*. A Unifying Approach for GFR Estimation: Recommendations of the NKF-ASN Task Force on Reassessing the Inclusion of Race in Diagnosing Kidney Disease. *Am J Kidney Dis*. 2022 Feb;79(2):268-288.e1. doi: 10.1053/j.ajkd.2021.08.003.

**4. Implications for removing the race correction factor in CKD-EPI equation:**

- Diao JA, Wu GJ, Taylor HA, *et al*. Clinical Implications of Removing Race From Estimates of Kidney Function. *JAMA*. 2021 Jan 12;325(2):184-186. doi: 10.1001/jama.2020.22124.
- Tsai JW, Cerdeña JP, Goedel WC, *et al*. Evaluating the Impact and Rationale of Race-Specific Estimations of Kidney Function: Estimations from U.S. NHANES, 2015-2018. *EClinicalMedicine*. 2021 Nov 19;42:101197. doi: 10.1016/j.eclinm.2021.101197.
- Ahmed S, Nutt CT, Eneanya ND, *et al*. Examining the Potential Impact of Race Multiplier Utilization in Estimated Glomerular Filtration Rate Calculation on African-American Care Outcomes. *J Gen Intern Med*. 2021 Feb;36(2):464-471. doi: 10.1007/s11606-020-06280-5.

**5. Policy changes aimed to combat race-based clinical algorithms:**

*Coalition to End Racism in Clinical Algorithms (CERCA) report*

- Clinical Algorithms (CERCA) - NYC Health [Internet]. www.nyc.gov. [cited 2023 Apr 11]. Available from: https://www.nyc.gov/site/doh/providers/resources/coalition-to-end-racism-in-clinical-algorithms.page

*Agency for Healthcare Research and Quality's (AHRQ) call for action*

- Warren, Wyden, Booker, and Lee question the use of race-based algorithms in Standard Medical Practice: U.S. senator Elizabeth Warren of Massachusetts [Internet]. Warren, Wyden, Booker, and Lee Question the Use of Race-Based Algorithms in Standard Medical Practice | U.S. Senator Elizabeth Warren of Massachusetts. 2020 [cited 2023 Apr11]. Available from: https://www.warren.senate.gov/newsroom/press-releases/warren-wyden-booker-and-lee-question-the-use-of-race-based-algorithms-in-standard-medical-practice

*AHRQ Protocol for Systematic review of Race-based clinical algorithms*

- Impact of Healthcare Algorithms on Racial and Ethnic Disparities in Health and Healthcare [Internet]. effectivehealthcare.ahrq.gov. Available from: https://effectivehealthcare.ahrq.gov/products/racial-disparities-health-healthcare/protocol

**6. Novel biomarkers to assess kidney function:**

- Ebert N, Schaeffner E. New biomarkers for estimating glomerular filtration rate. *J Lab Precis Med.* 2018;3:75.

**7. Patient perspective on race-based algorithms:**

- Schmidt, I.M., Shohet, M., Serrano, M. *et al.* Patients’ Perspectives on Race and the Use of Race-Based Algorithms in Clinical Decision-Making: a Qualitative Study. *J GEN INTERN MED*. 2023 Feb 22:1-7. https://doi.org/10.1007/s11606-023-08035-4
