# Supplementary material for: Reassessing the Use of Race in Clinical Algorithms: An Interactive, Case-Based Session for Medical Students Using eGFR
Source: MedEdPORTAL. 2024 Jun 21;20:11412. doi: 10.15766/mep_2374-8265.11412 (PMC11219082; doi:10.15766/mep_2374-8265.11412)
Supplement: Supplementary file 1 — Presentation.pptxFacilitator Guide.docxEvaluation Forms.docxResources for Interested Students.docx [file mep_2374-8265.11412-s001.zip › A. Presentation.pptx]

## Slide 1
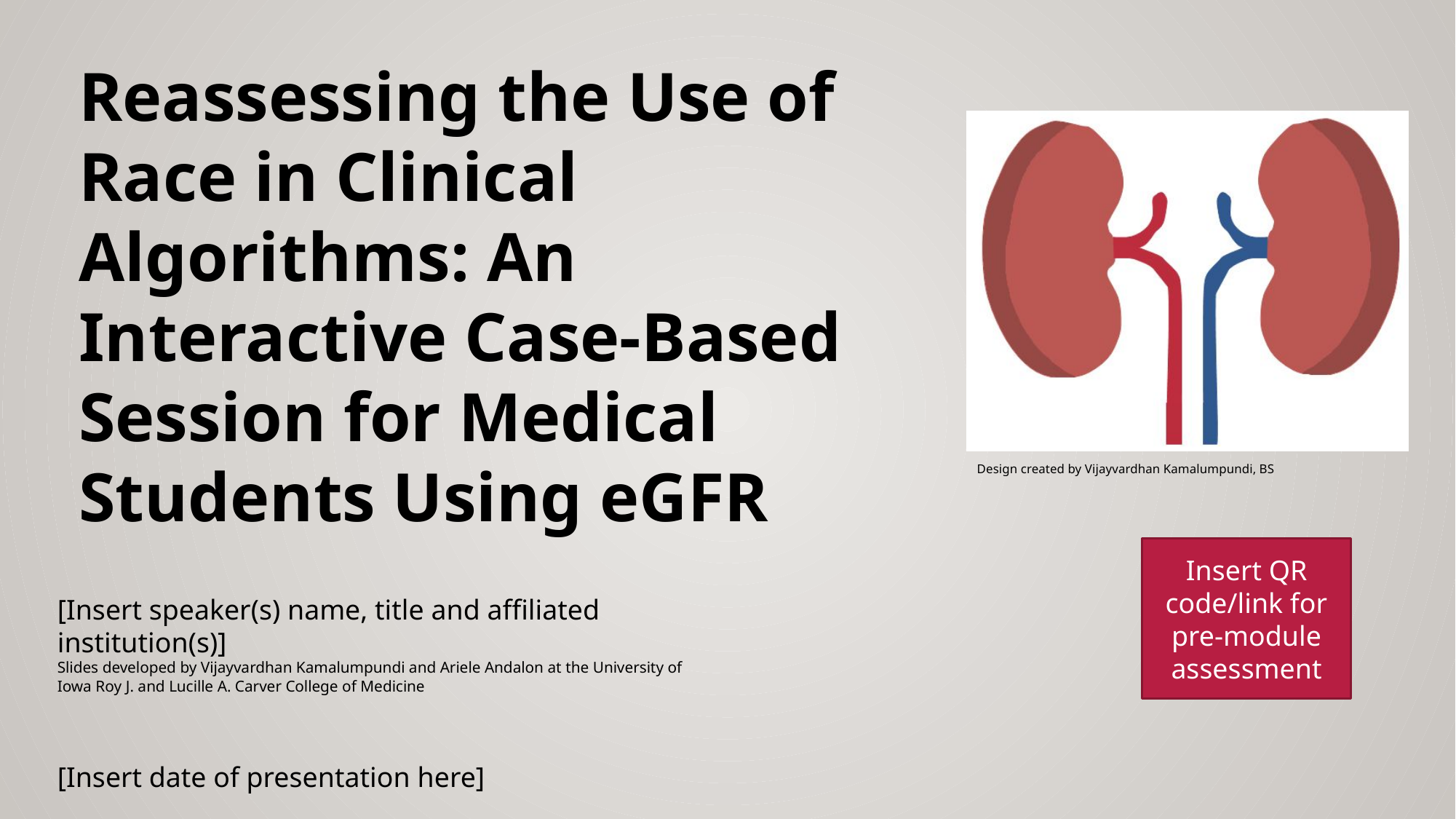

Reassessing the Use of Race in Clinical Algorithms: An Interactive Case-Based Session for Medical Students Using eGFR
Design created by Vijayvardhan Kamalumpundi, BS
Insert QR code/link for pre-module assessment
[Insert speaker(s) name, title and affiliated institution(s)]
Slides developed by Vijayvardhan Kamalumpundi and Ariele Andalon at the University of Iowa Roy J. and Lucille A. Carver College of Medicine
[Insert date of presentation here]

## Slide 2
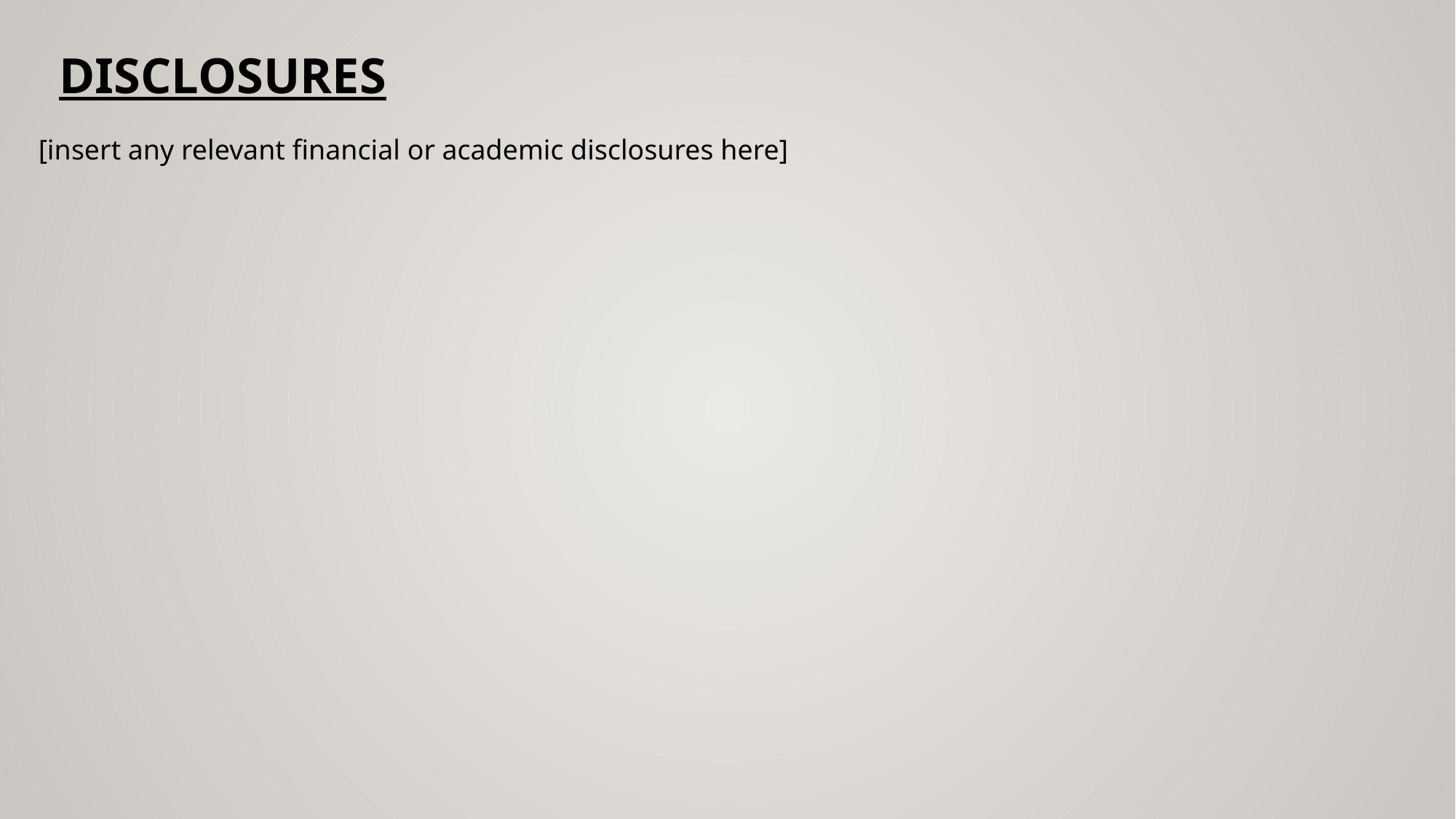

Disclosures
[insert any relevant financial or academic disclosures here]

## Slide 3
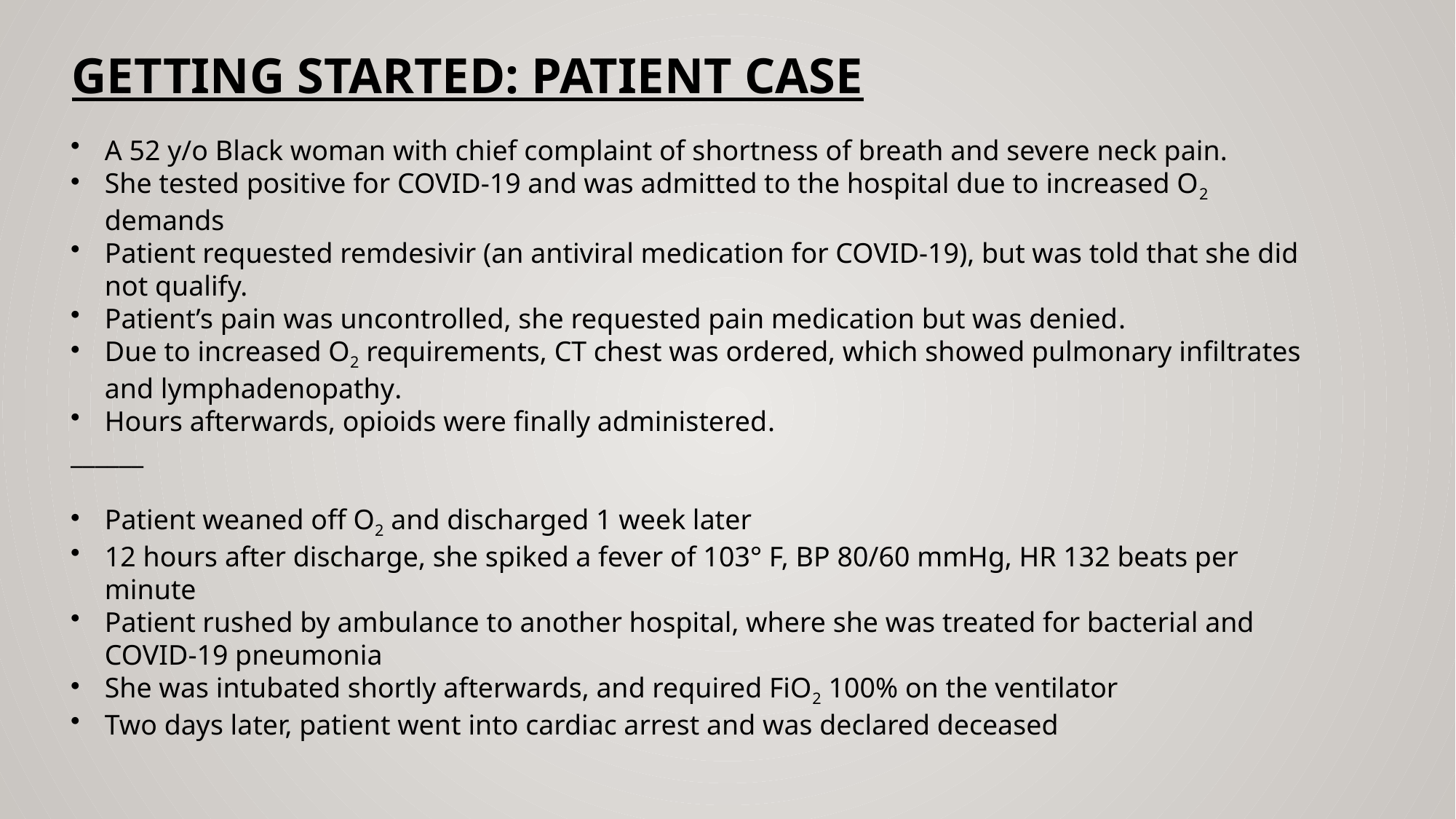

Getting started: Patient Case
A 52 y/o Black woman with chief complaint of shortness of breath and severe neck pain.
She tested positive for COVID-19 and was admitted to the hospital due to increased O2 demands
Patient requested remdesivir (an antiviral medication for COVID-19), but was told that she did not qualify.
Patient’s pain was uncontrolled, she requested pain medication but was denied.
Due to increased O2 requirements, CT chest was ordered, which showed pulmonary infiltrates and lymphadenopathy.
Hours afterwards, opioids were finally administered.
______
Patient weaned off O2 and discharged 1 week later
12 hours after discharge, she spiked a fever of 103° F, BP 80/60 mmHg, HR 132 beats per minute
Patient rushed by ambulance to another hospital, where she was treated for bacterial and COVID-19 pneumonia
She was intubated shortly afterwards, and required FiO2 100% on the ventilator
Two days later, patient went into cardiac arrest and was declared deceased

## Slide 4
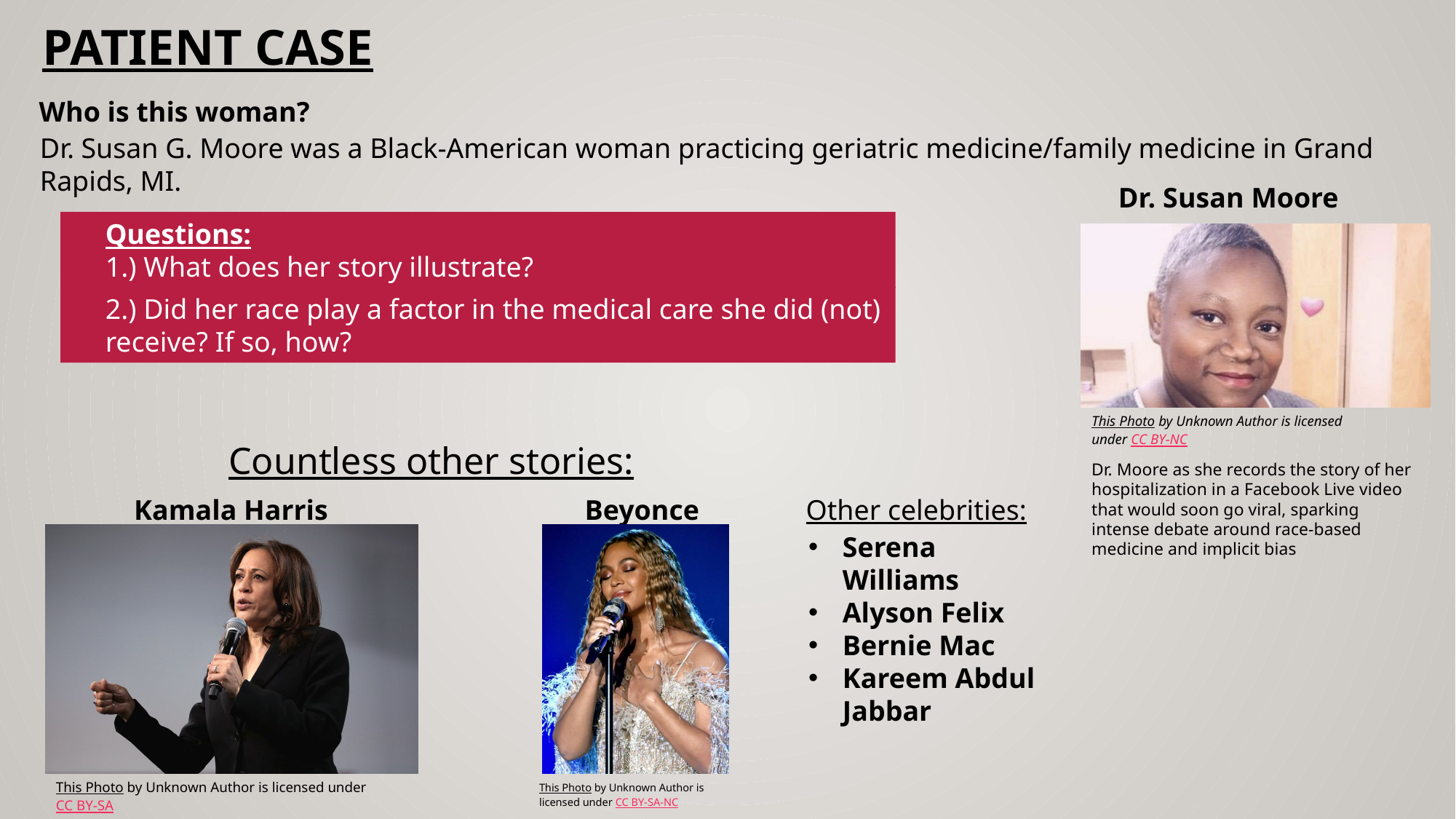

Patient Case
Who is this woman?
Dr. Susan G. Moore was a Black-American woman practicing geriatric medicine/family medicine in Grand Rapids, MI.
Dr. Susan Moore
Questions:
1.) What does her story illustrate?
2.) Did her race play a factor in the medical care she did (not) receive? If so, how?
This Photo by Unknown Author is licensed under CC BY-NC
Countless other stories:
Dr. Moore as she records the story of her hospitalization in a Facebook Live video that would soon go viral, sparking intense debate around race-based medicine and implicit bias
Kamala Harris
Beyonce
Other celebrities:
Serena Williams
Alyson Felix
Bernie Mac
Kareem Abdul Jabbar
This Photo by Unknown Author is licensed under CC BY-SA
This Photo by Unknown Author is licensed under CC BY-SA-NC

## Slide 5
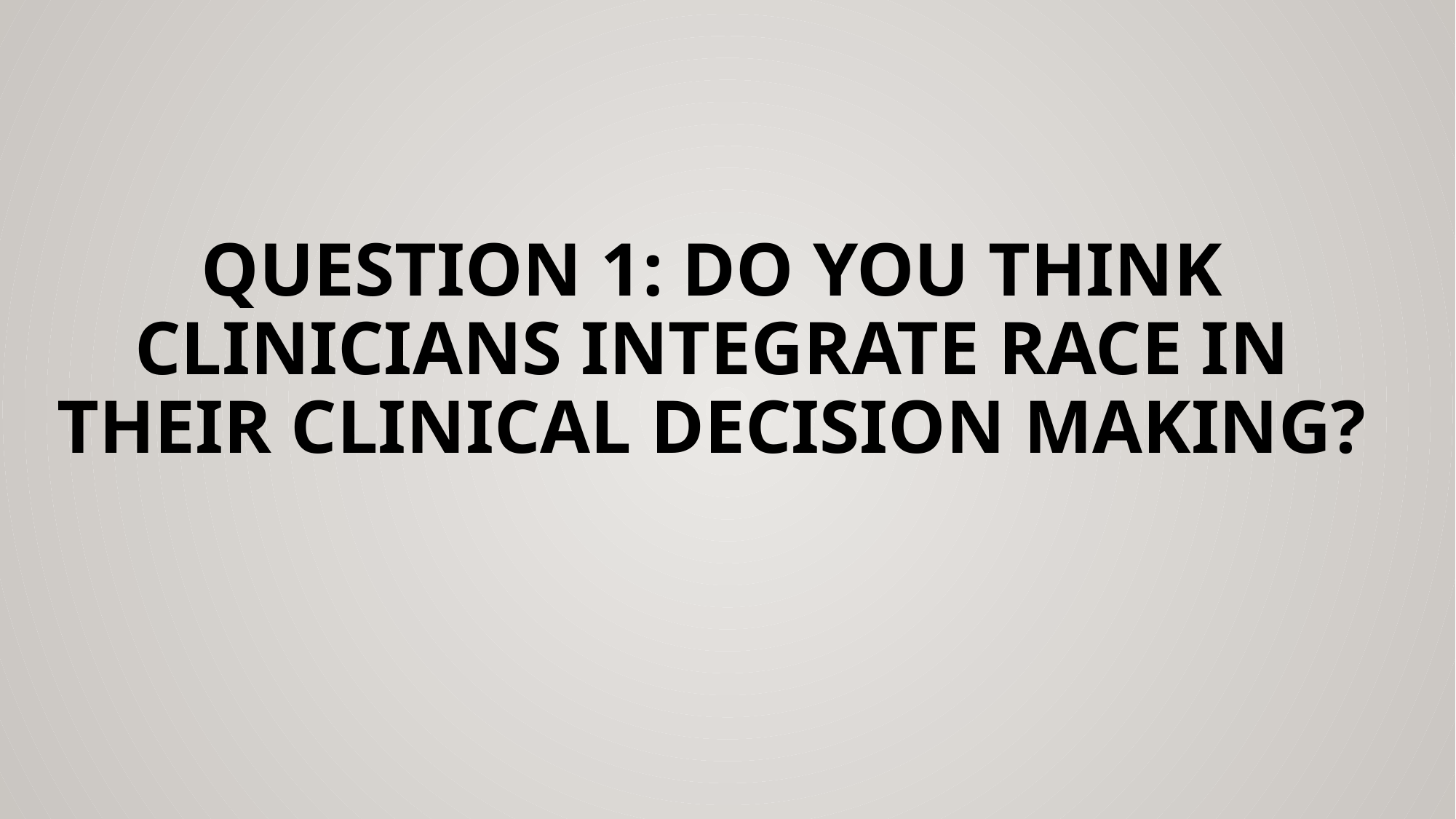

Question 1: Do you think clinicians integrate race in their clinical decision making?

## Slide 6
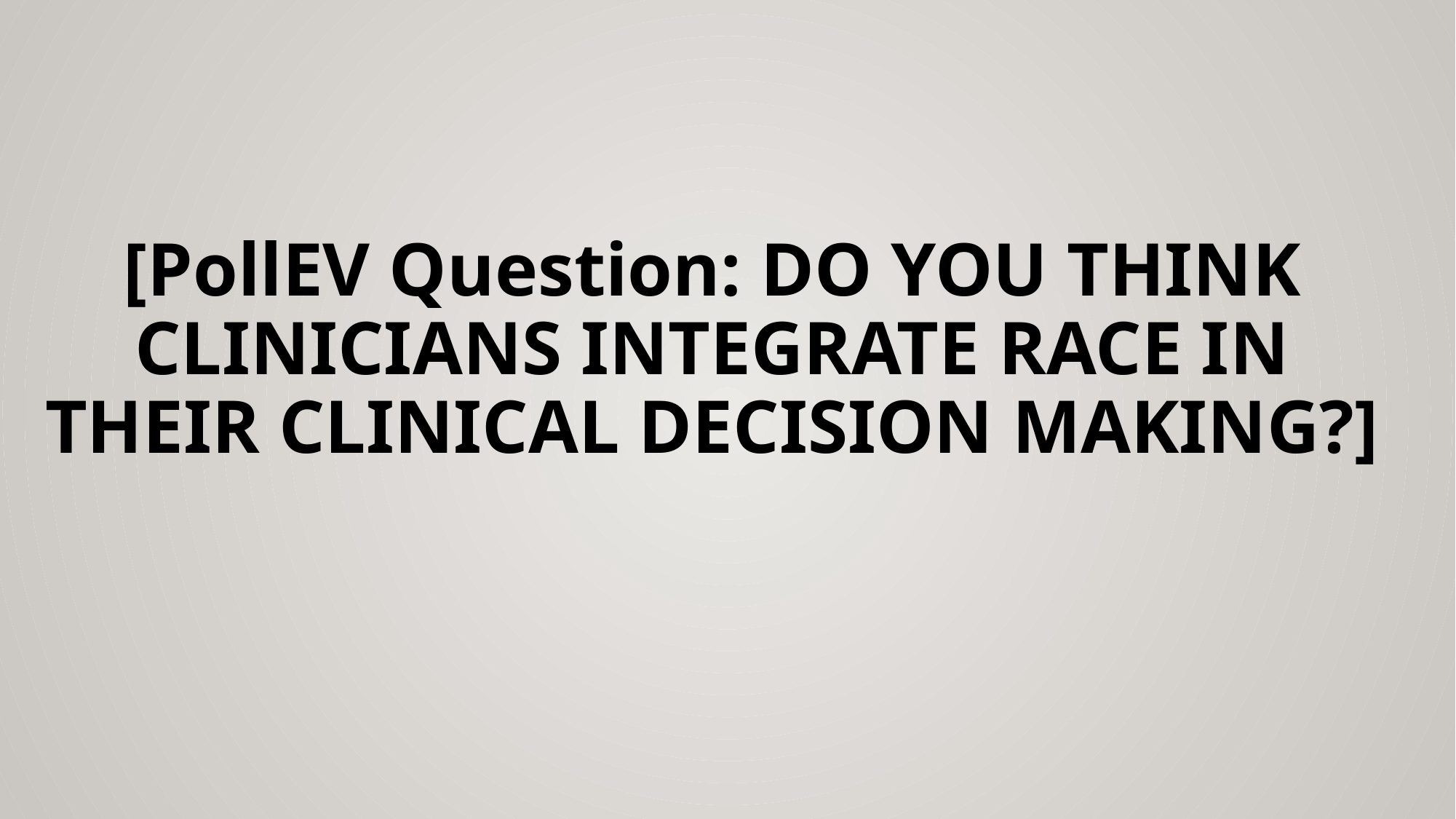

[pollev question: Do you think clinicians integrate race in their clinical decision making?]

## Slide 7
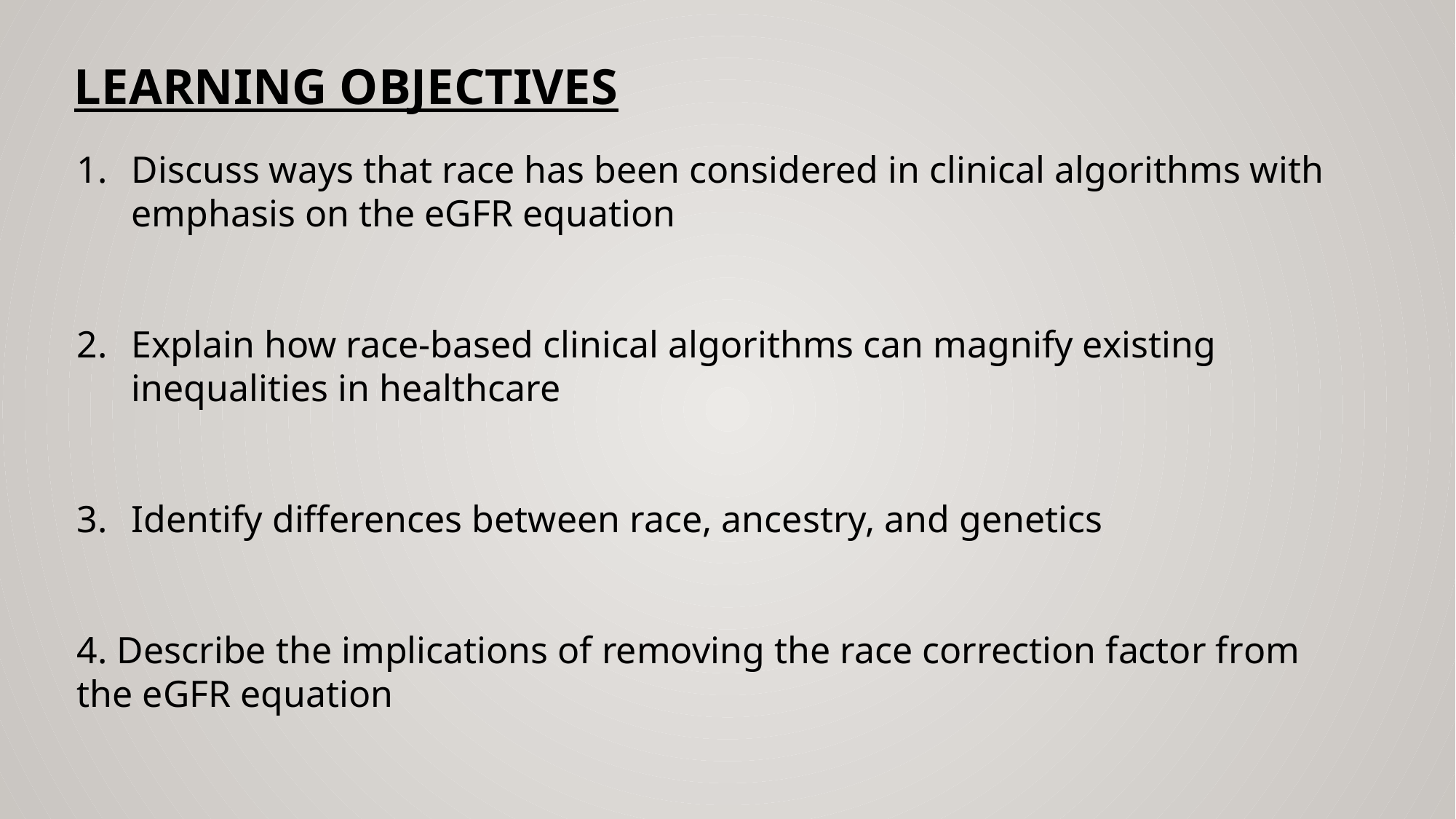

Learning Objectives
Discuss ways that race has been considered in clinical algorithms with emphasis on the eGFR equation
Explain how race-based clinical algorithms can magnify existing inequalities in healthcare
Identify differences between race, ancestry, and genetics
4. Describe the implications of removing the race correction factor from the eGFR equation

## Slide 8
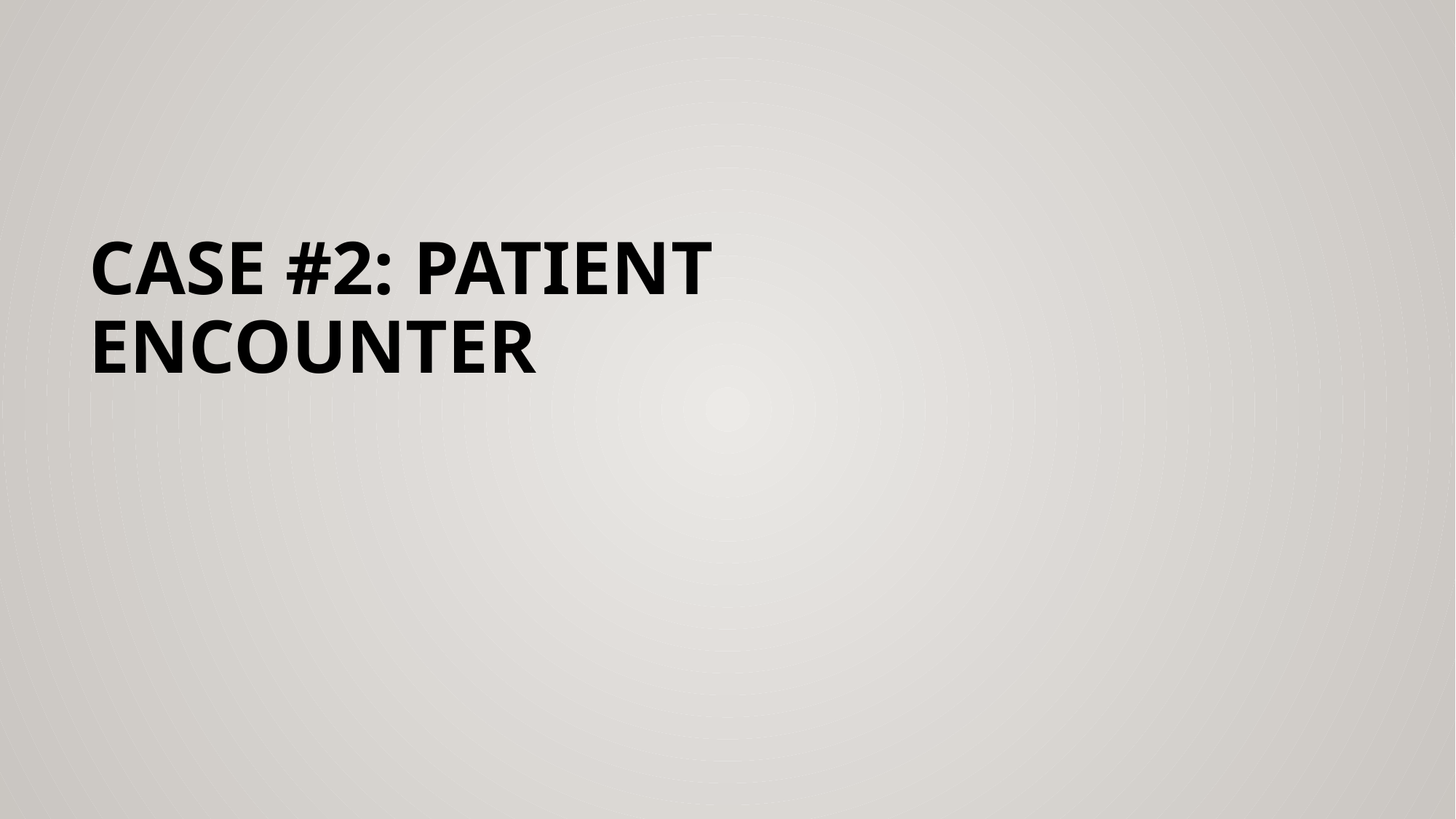

Case #2: Patient Encounter

## Slide 9
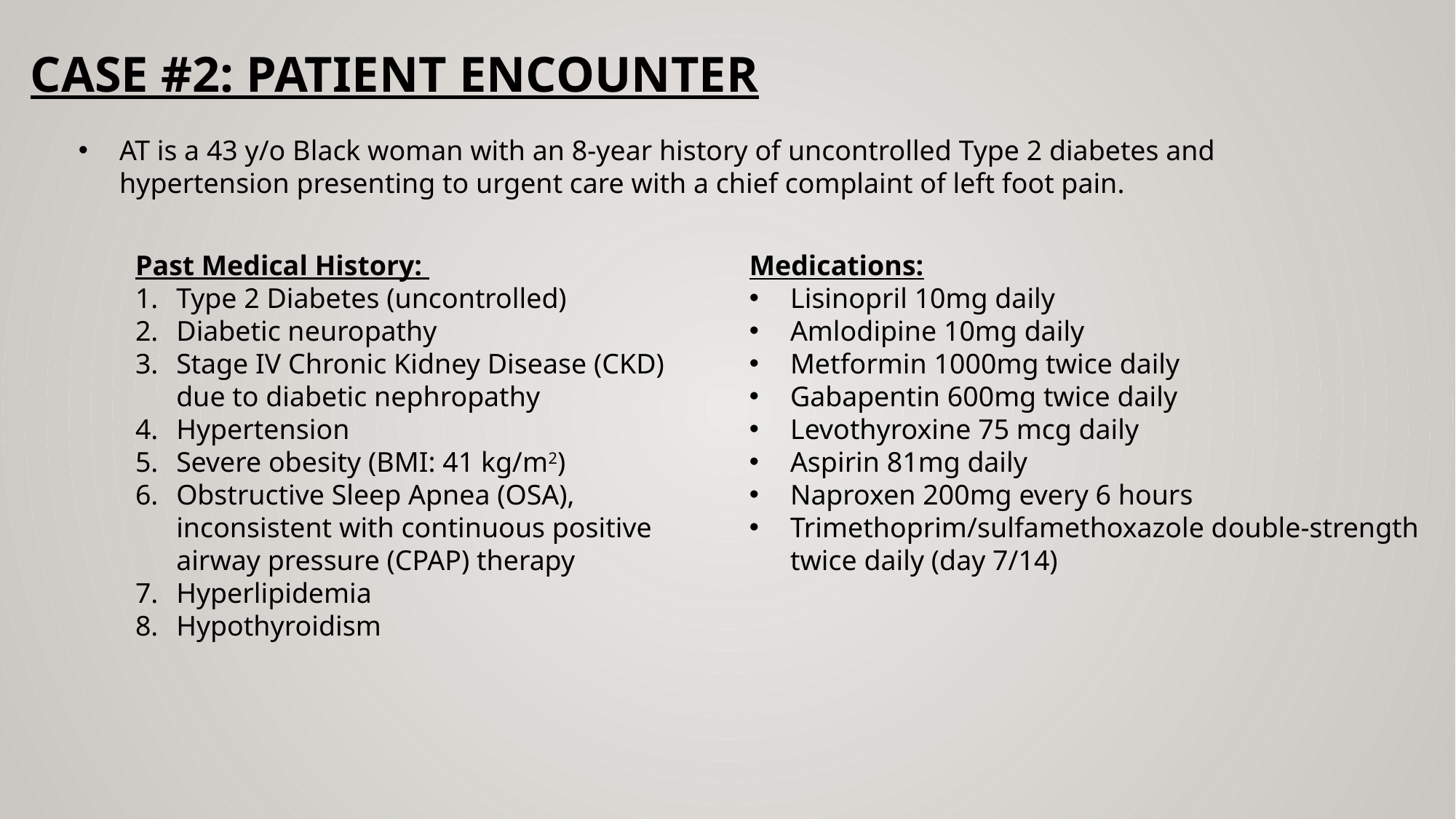

Case #2: Patient Encounter
AT is a 43 y/o Black woman with an 8-year history of uncontrolled Type 2 diabetes and hypertension presenting to urgent care with a chief complaint of left foot pain.
Past Medical History:
Type 2 Diabetes (uncontrolled)
Diabetic neuropathy
Stage IV Chronic Kidney Disease (CKD) due to diabetic nephropathy
Hypertension
Severe obesity (BMI: 41 kg/m2)
Obstructive Sleep Apnea (OSA), inconsistent with continuous positive airway pressure (CPAP) therapy
Hyperlipidemia
Hypothyroidism
Medications:
Lisinopril 10mg daily
Amlodipine 10mg daily
Metformin 1000mg twice daily
Gabapentin 600mg twice daily
Levothyroxine 75 mcg daily
Aspirin 81mg daily
Naproxen 200mg every 6 hours
Trimethoprim/sulfamethoxazole double-strength twice daily (day 7/14)

## Slide 10
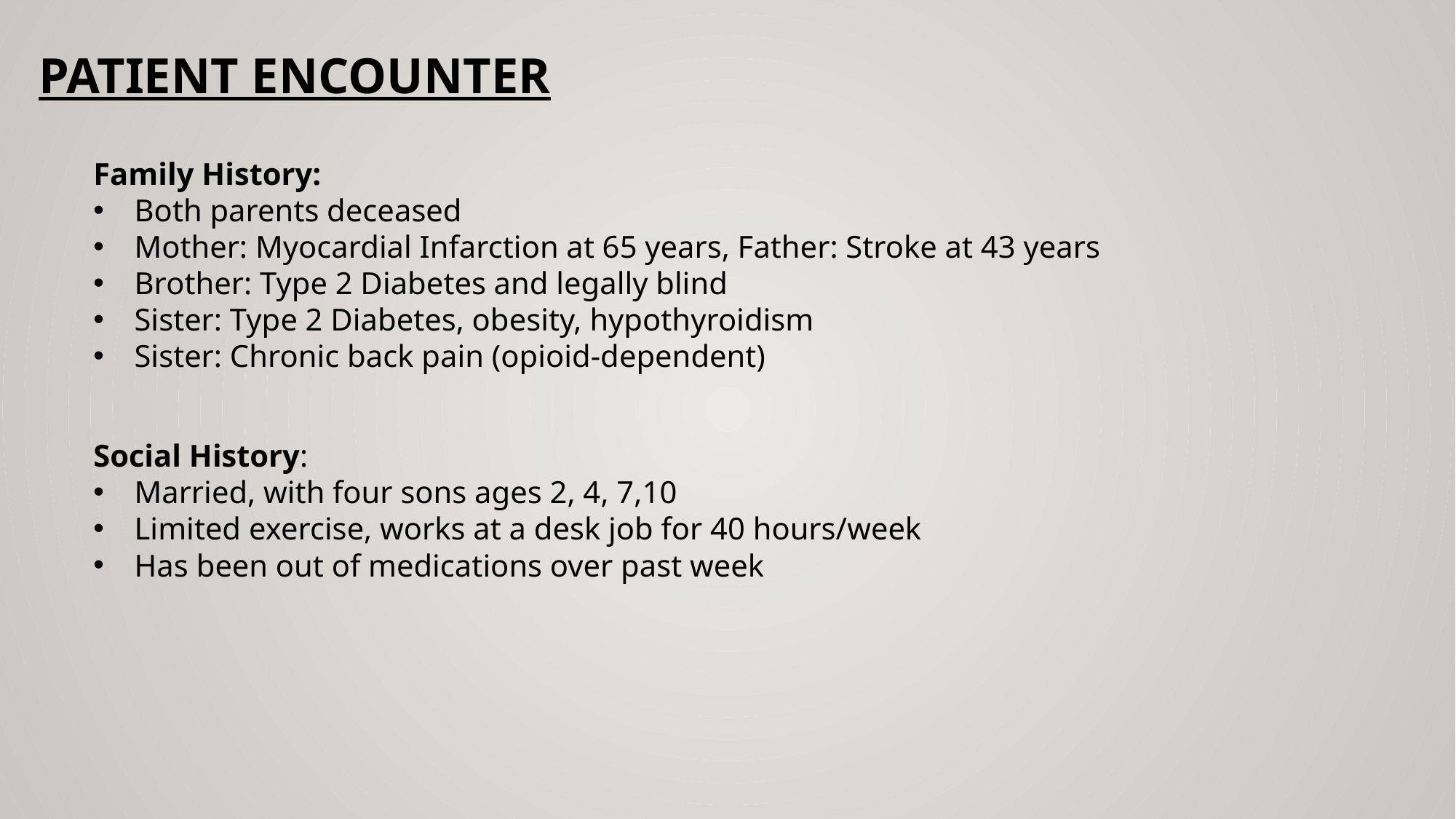

Patient Encounter
Family History:
Both parents deceased
Mother: Myocardial Infarction at 65 years, Father: Stroke at 43 years
Brother: Type 2 Diabetes and legally blind
Sister: Type 2 Diabetes, obesity, hypothyroidism
Sister: Chronic back pain (opioid-dependent)
Social History:
Married, with four sons ages 2, 4, 7,10
Limited exercise, works at a desk job for 40 hours/week
Has been out of medications over past week

## Slide 11
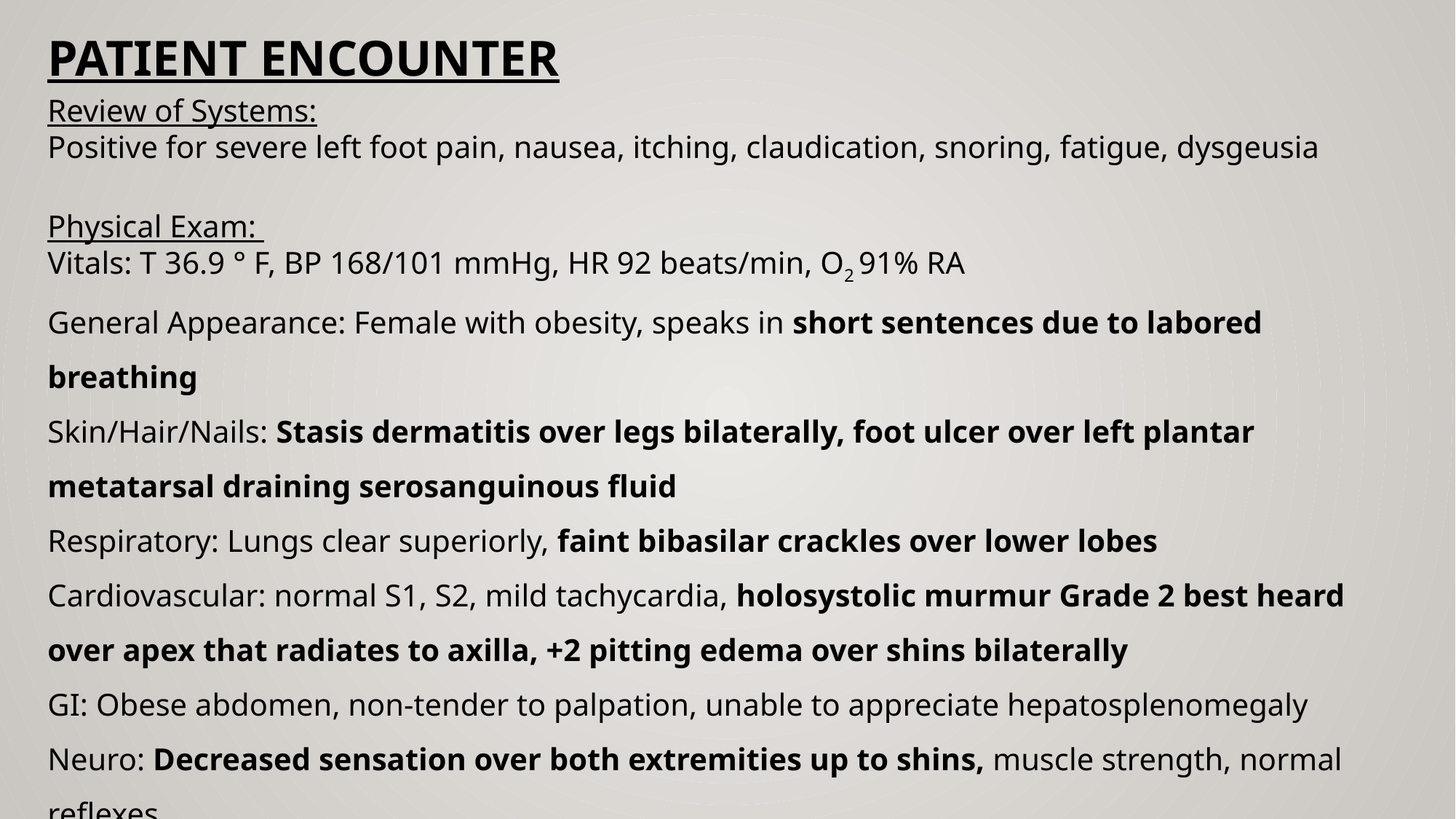

Patient Encounter
Review of Systems:
Positive for severe left foot pain, nausea, itching, claudication, snoring, fatigue, dysgeusia
Physical Exam:
Vitals: T 36.9 ° F, BP 168/101 mmHg, HR 92 beats/min, O2 91% RA
General Appearance: Female with obesity, speaks in short sentences due to labored breathing
Skin/Hair/Nails: Stasis dermatitis over legs bilaterally, foot ulcer over left plantar metatarsal draining serosanguinous fluid
Respiratory: Lungs clear superiorly, faint bibasilar crackles over lower lobes
Cardiovascular: normal S1, S2, mild tachycardia, holosystolic murmur Grade 2 best heard over apex that radiates to axilla, +2 pitting edema over shins bilaterally
GI: Obese abdomen, non-tender to palpation, unable to appreciate hepatosplenomegaly
Neuro: Decreased sensation over both extremities up to shins, muscle strength, normal reflexes

## Slide 12
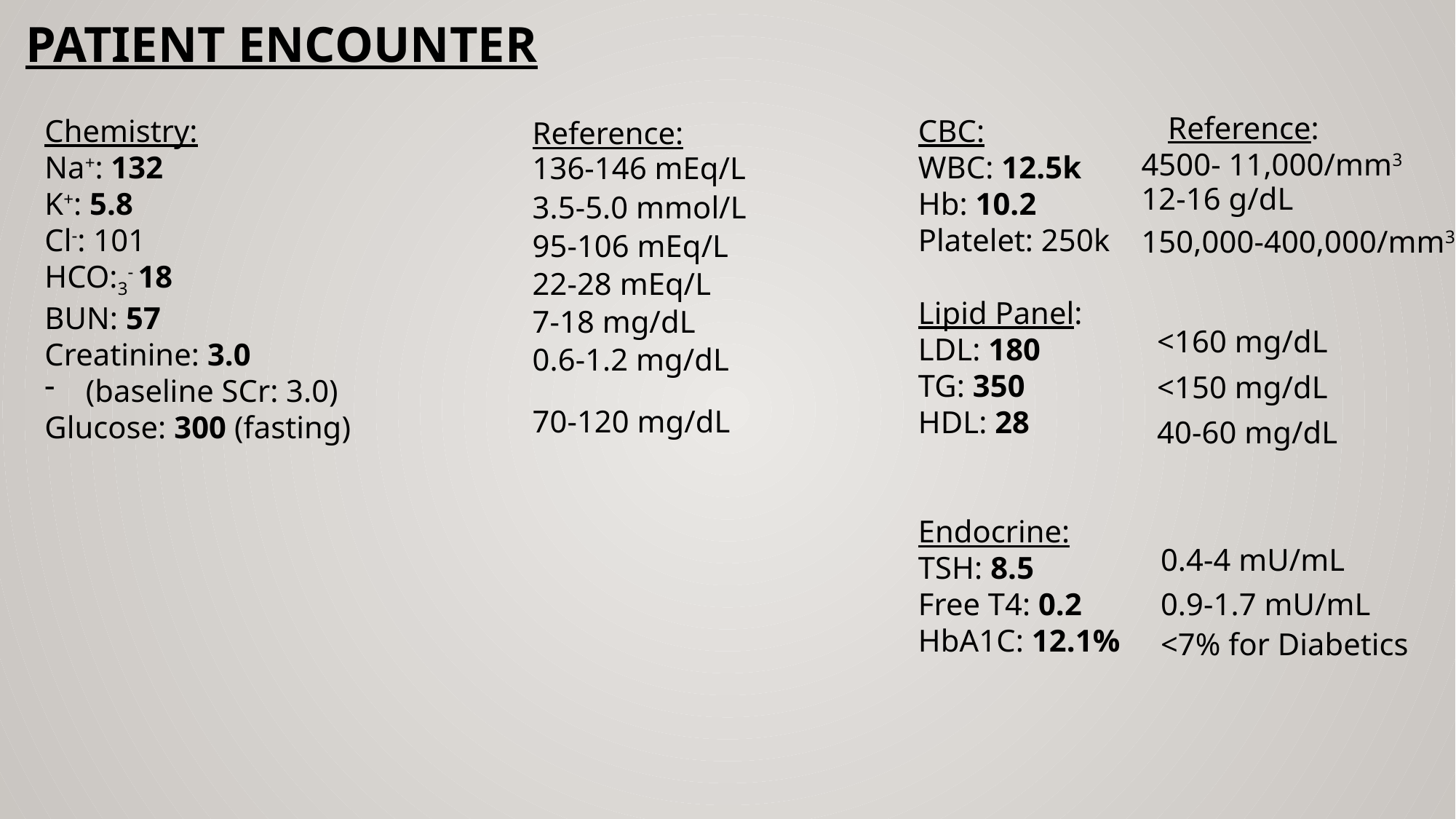

Patient Encounter
Reference:
Chemistry:
Na+: 132
K+: 5.8
Cl-: 101
HCO:3- 18
BUN: 57
Creatinine: 3.0
(baseline SCr: 3.0)
Glucose: 300 (fasting)
CBC:
WBC: 12.5k
Hb: 10.2
Platelet: 250k
Lipid Panel:
LDL: 180
TG: 350
HDL: 28
Endocrine:
TSH: 8.5
Free T4: 0.2
HbA1C: 12.1%
Reference:
4500- 11,000/mm3
136-146 mEq/L
12-16 g/dL
3.5-5.0 mmol/L
150,000-400,000/mm3
95-106 mEq/L
22-28 mEq/L
7-18 mg/dL
<160 mg/dL
0.6-1.2 mg/dL
<150 mg/dL
70-120 mg/dL
40-60 mg/dL
0.4-4 mU/mL
0.9-1.7 mU/mL
<7% for Diabetics

## Slide 13
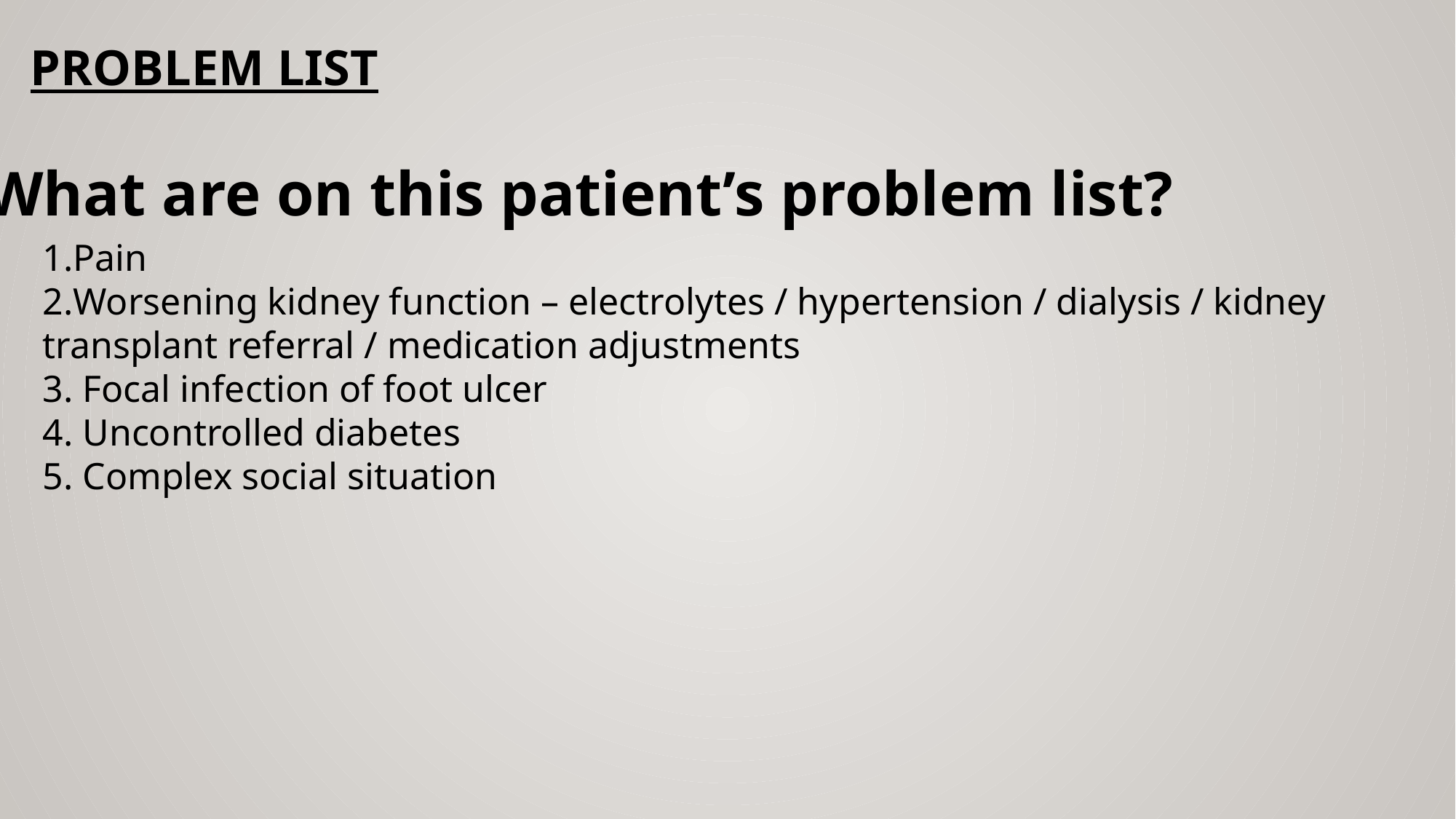

Problem list
What are on this patient’s problem list?
Pain
Worsening kidney function – electrolytes / hypertension / dialysis / kidney transplant referral / medication adjustments
3. Focal infection of foot ulcer
4. Uncontrolled diabetes
5. Complex social situation

## Slide 14
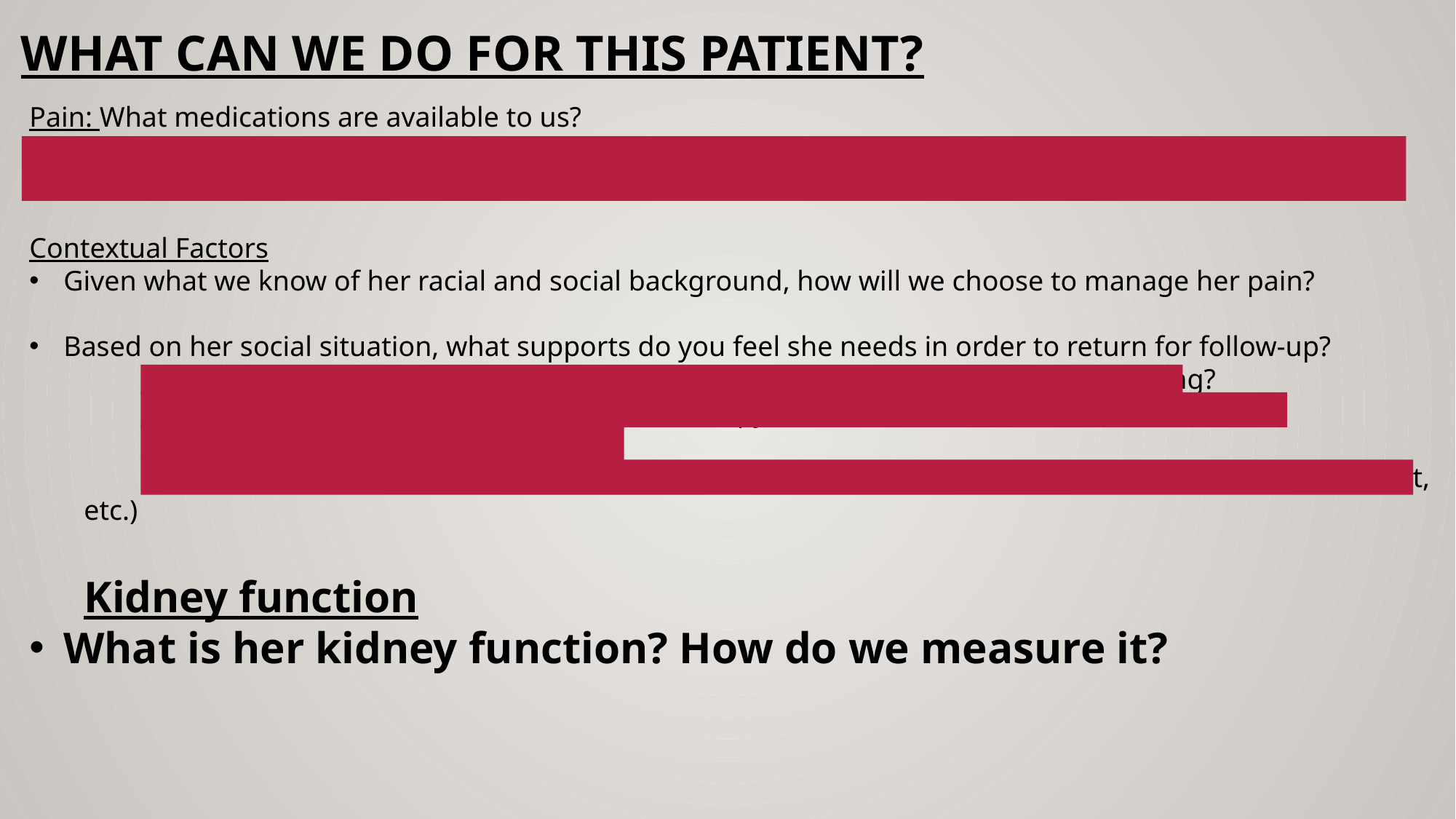

What can we do for this patient?
Pain: What medications are available to us?
 Non-steroidal anti-inflammatory medications (Ibuprofen, Naproxen), acetaminophen, opioids, gabapentin/pregabalin, tricyclic antidepressants, duloxetine what do we prefer here?
Contextual Factors
Given what we know of her racial and social background, how will we choose to manage her pain?
Based on her social situation, what supports do you feel she needs in order to return for follow-up?
 - Mental Health: Is there possibly an element of anxiety/depression we are missing?
 - Should we involve social work, physical therapy?
 - Insurance issues?
 - Other structural barriers that are preventing care (e.g. lack of transportation, lack of social support, etc.)
Kidney function
What is her kidney function? How do we measure it?

## Slide 15
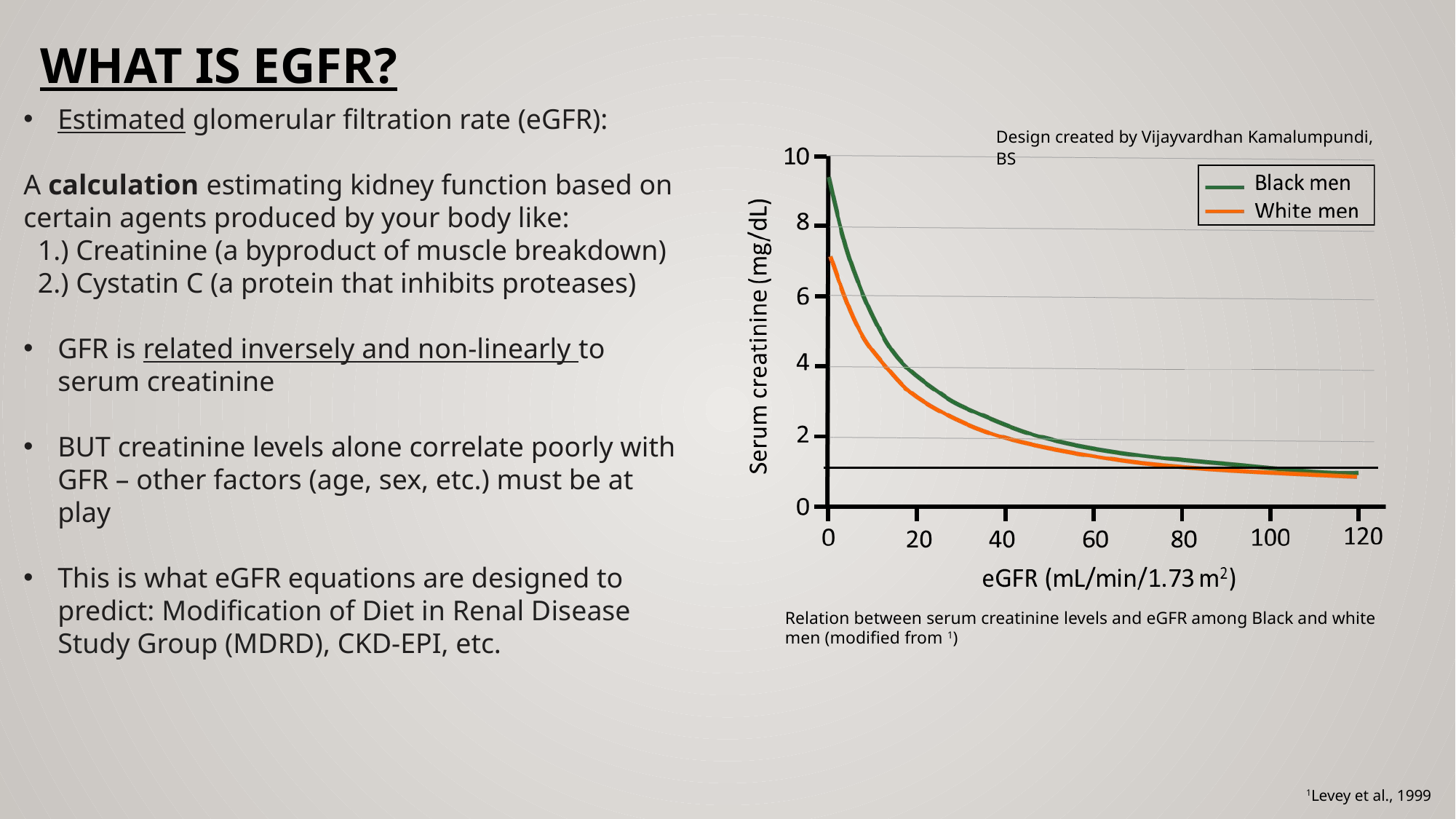

What is eGFR?
Estimated glomerular filtration rate (eGFR):
A calculation estimating kidney function based on certain agents produced by your body like:
 1.) Creatinine (a byproduct of muscle breakdown)
 2.) Cystatin C (a protein that inhibits proteases)
GFR is related inversely and non-linearly to serum creatinine
BUT creatinine levels alone correlate poorly with GFR – other factors (age, sex, etc.) must be at play
This is what eGFR equations are designed to predict: Modification of Diet in Renal Disease Study Group (MDRD), CKD-EPI, etc.
Design created by Vijayvardhan Kamalumpundi, BS
Relation between serum creatinine levels and eGFR among Black and white men (modified from 1)
1Levey et al., 1999

## Slide 16
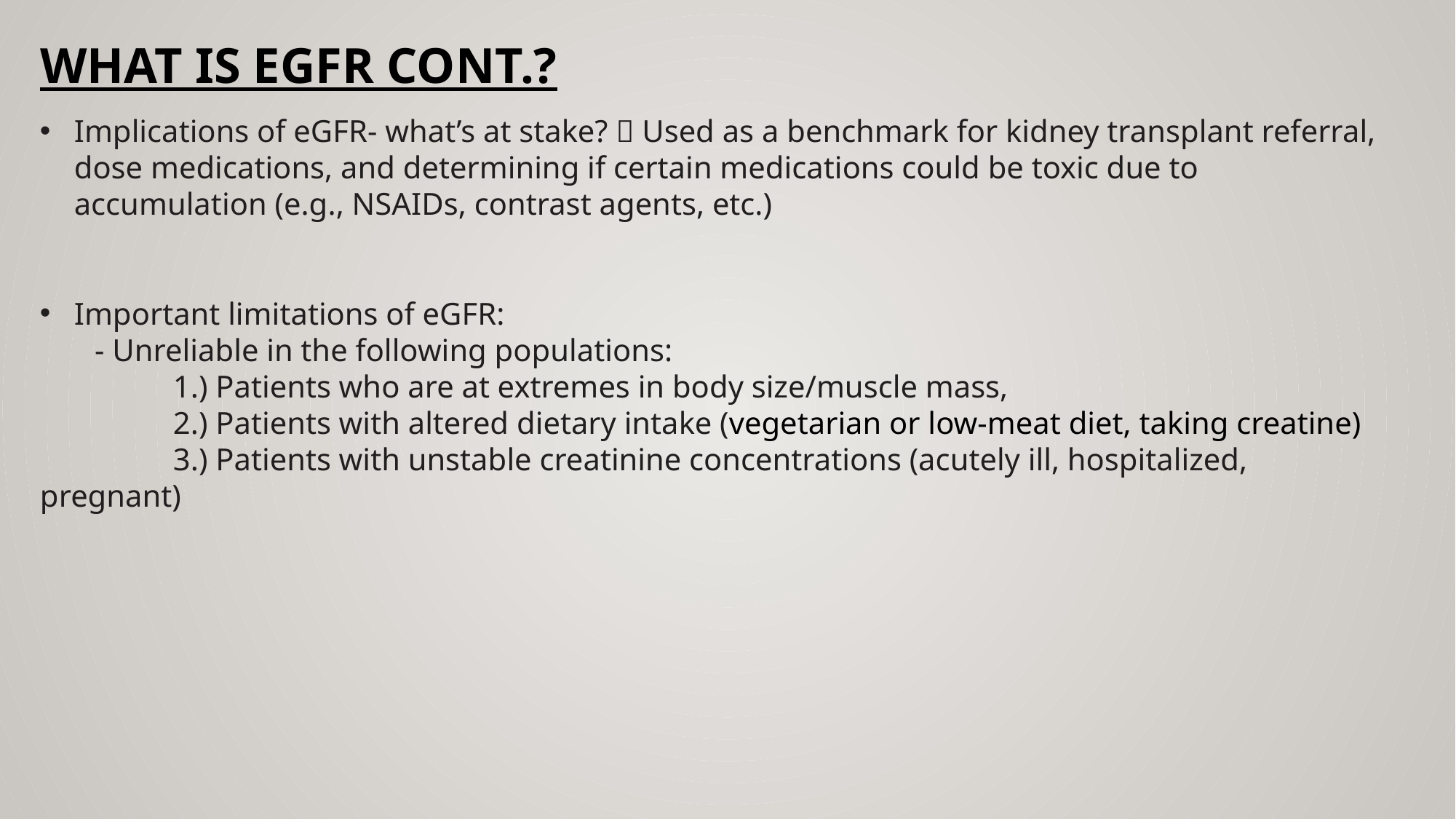

What is eGFR cont.?
Implications of eGFR- what’s at stake?  Used as a benchmark for kidney transplant referral, dose medications, and determining if certain medications could be toxic due to accumulation (e.g., NSAIDs, contrast agents, etc.)
Important limitations of eGFR:
 - Unreliable in the following populations:
 1.) Patients who are at extremes in body size/muscle mass,
 2.) Patients with altered dietary intake (vegetarian or low-meat diet, taking creatine)
 3.) Patients with unstable creatinine concentrations (acutely ill, hospitalized, pregnant)

## Slide 17
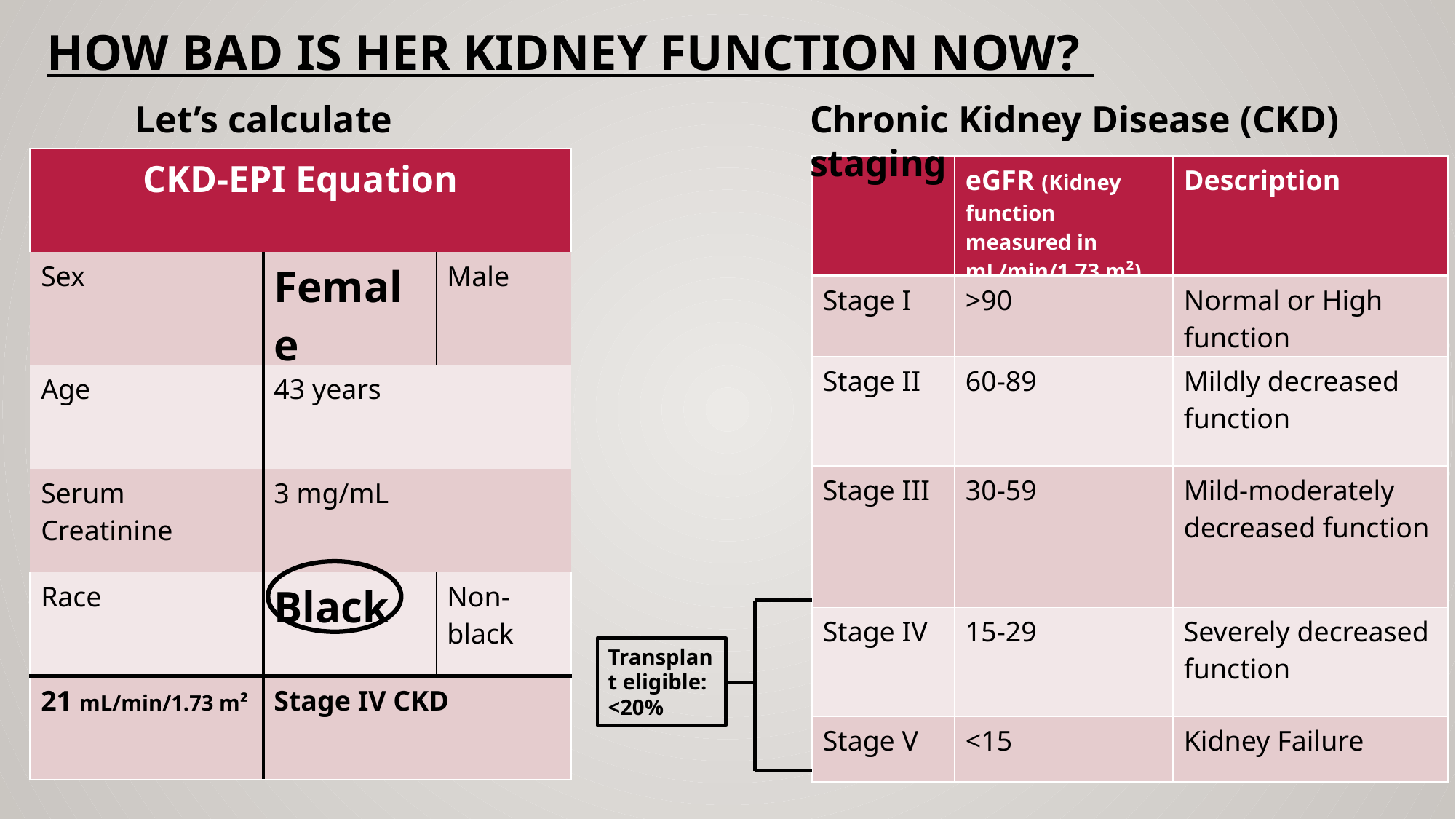

How bad is her kidney function now?
Let’s calculate it!
Chronic Kidney Disease (CKD) staging
| CKD-EPI Equation | | |
| --- | --- | --- |
| Sex | Female | Male |
| Age | 43 years | |
| Serum Creatinine | 3 mg/mL | |
| Race | Black | Non-black |
| 21 mL/min/1.73 m² | Stage IV CKD | |
| | eGFR (Kidney function measured in mL/min/1.73 m²) | Description |
| --- | --- | --- |
| Stage I | >90 | Normal or High function |
| Stage II | 60-89 | Mildly decreased function |
| Stage III | 30-59 | Mild-moderately decreased function |
| Stage IV | 15-29 | Severely decreased function |
| Stage V | <15 | Kidney Failure |
Transplant eligible: <20%

## Slide 18
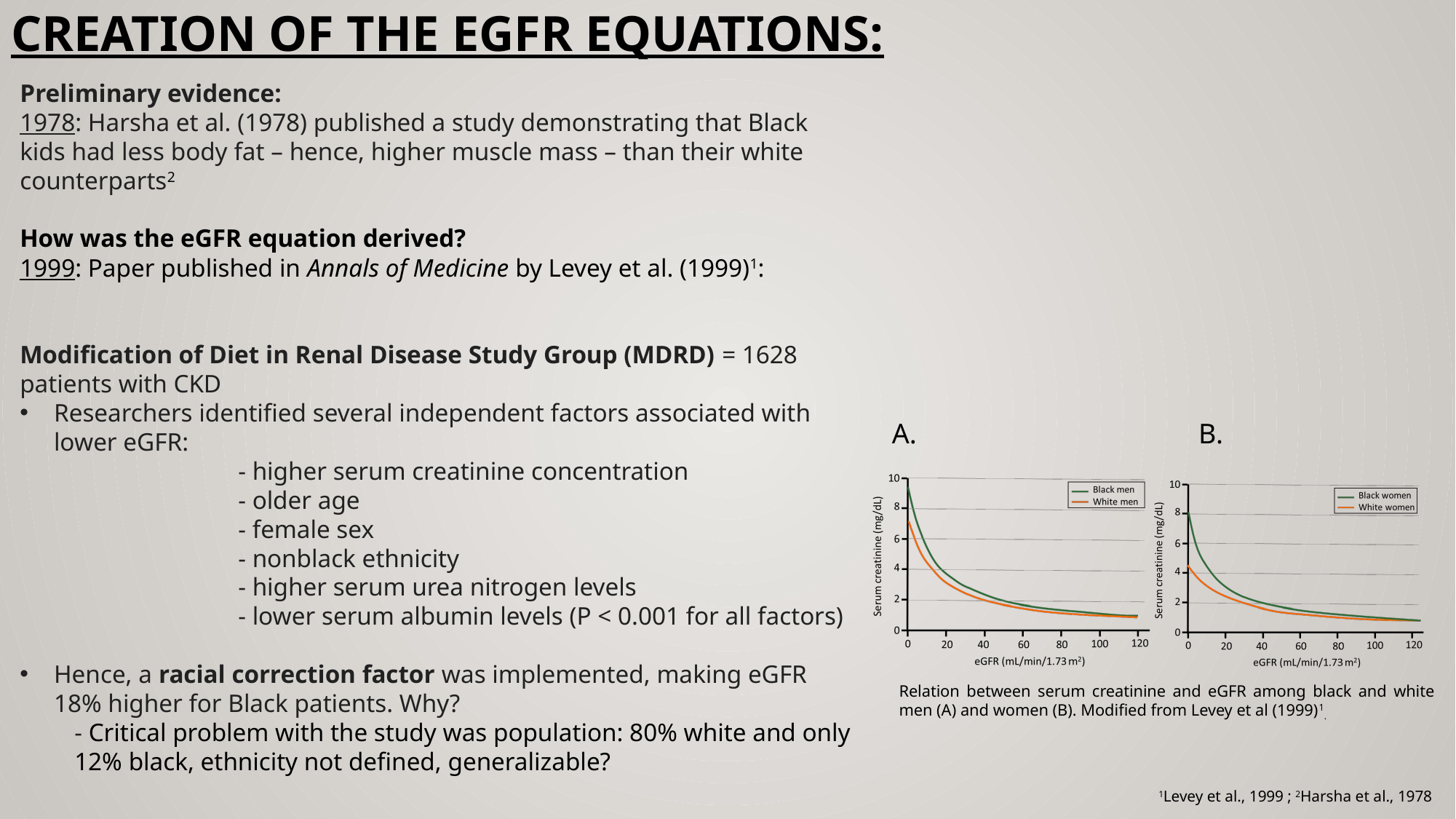

Creation of the eGFR equations:
Preliminary evidence:
1978: Harsha et al. (1978) published a study demonstrating that Black kids had less body fat – hence, higher muscle mass – than their white counterparts2
How was the eGFR equation derived?
1999: Paper published in Annals of Medicine by Levey et al. (1999)1:
Modification of Diet in Renal Disease Study Group (MDRD) = 1628 patients with CKD
Researchers identified several independent factors associated with lower eGFR:
	- higher serum creatinine concentration
	- older age
	- female sex
	- nonblack ethnicity
 	- higher serum urea nitrogen levels
	- lower serum albumin levels (P < 0.001 for all factors)
Hence, a racial correction factor was implemented, making eGFR 18% higher for Black patients. Why?
- Critical problem with the study was population: 80% white and only 12% black, ethnicity not defined, generalizable?
A.
B.
Relation between serum creatinine and eGFR among black and white men (A) and women (B). Modified from Levey et al (1999)1.
1Levey et al., 1999 ; 2Harsha et al., 1978

## Slide 19
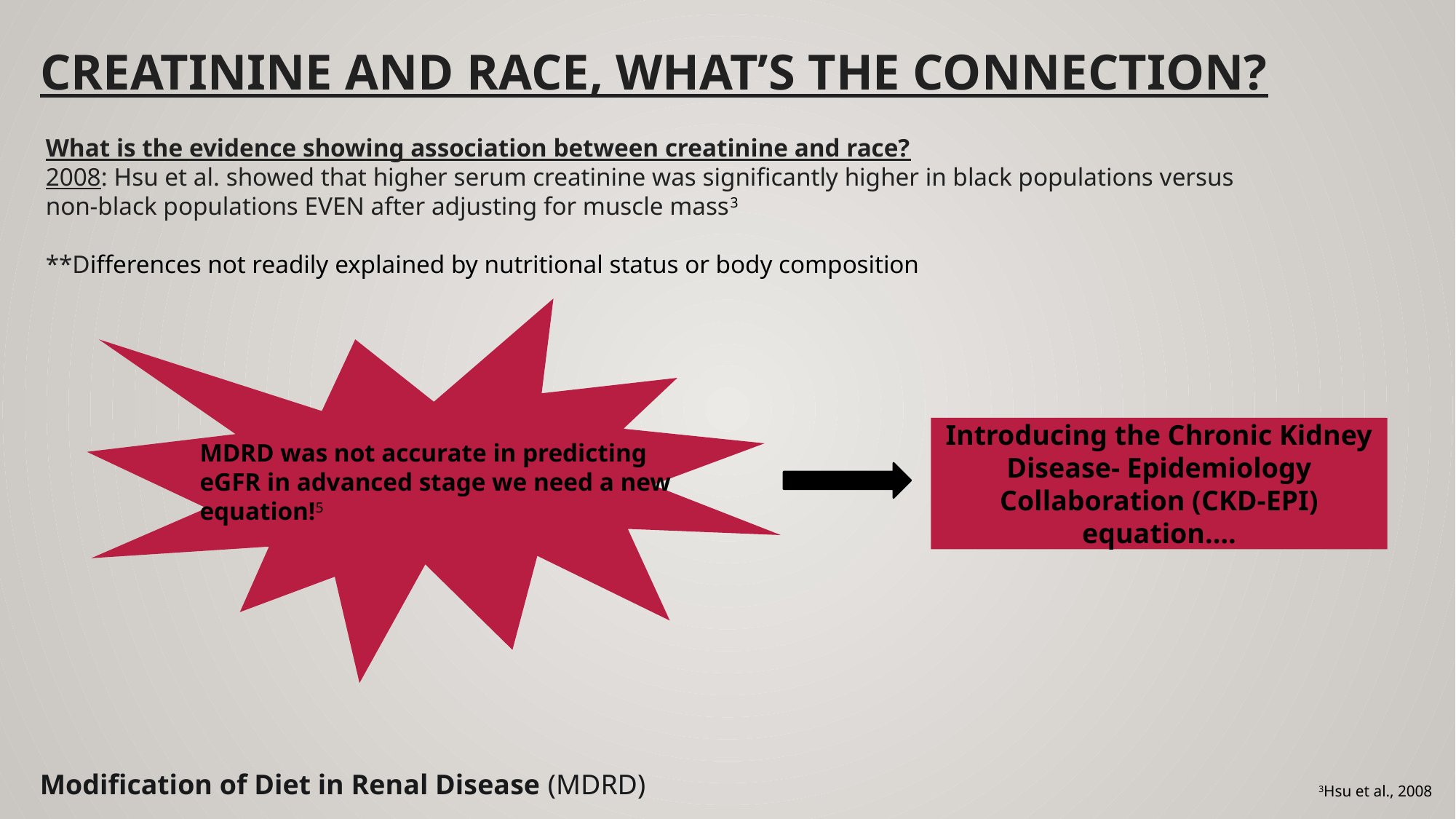

Creatinine and Race, what’s the connection?
What is the evidence showing association between creatinine and race?
2008: Hsu et al. showed that higher serum creatinine was significantly higher in black populations versus non-black populations EVEN after adjusting for muscle mass3
**Differences not readily explained by nutritional status or body composition
MDRD was not accurate in predicting eGFR in advanced stage we need a new equation!5
Introducing the Chronic Kidney Disease- Epidemiology Collaboration (CKD-EPI) equation….
Modification of Diet in Renal Disease (MDRD)
3Hsu et al., 2008

## Slide 20
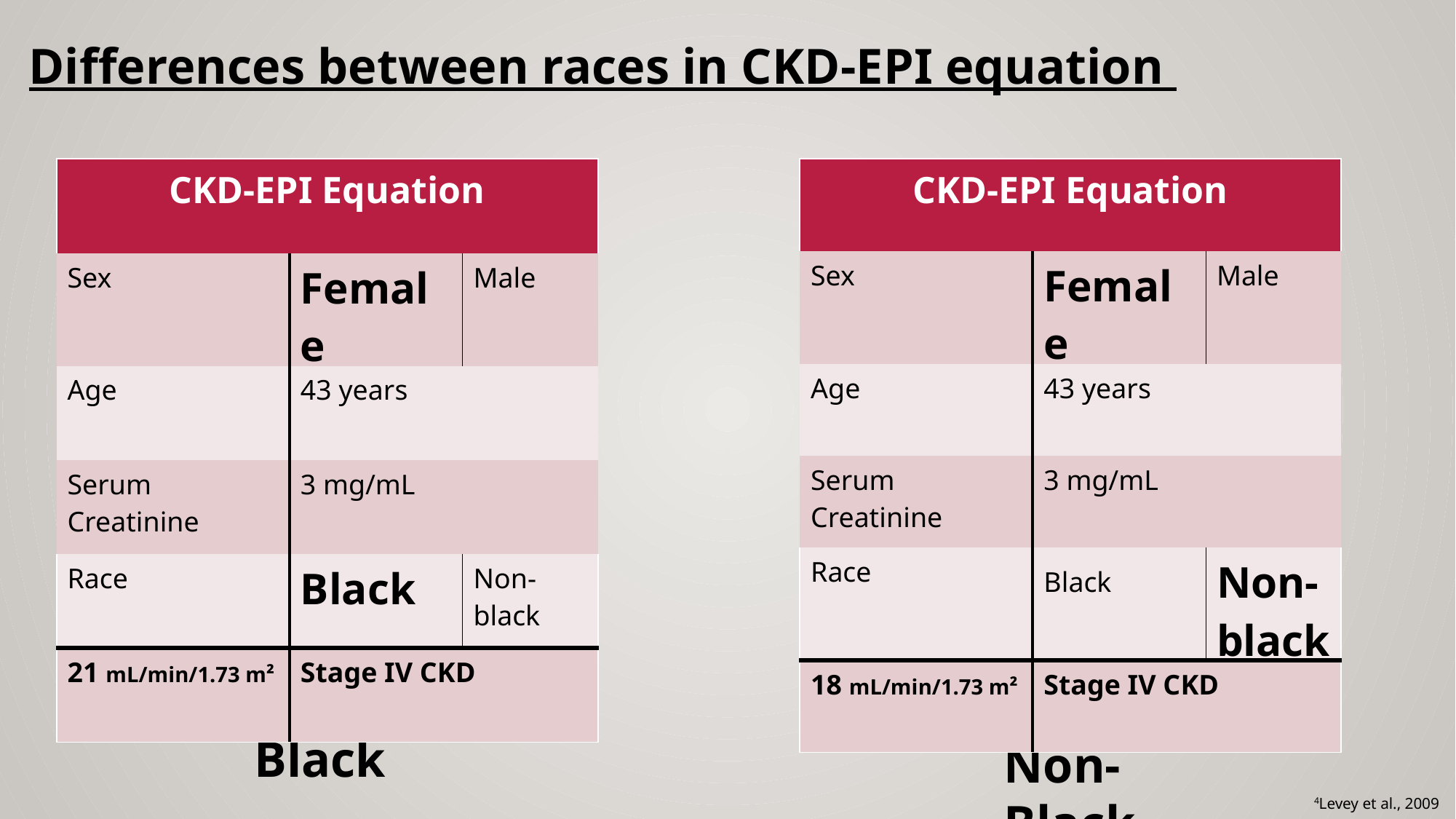

Differences between races in CKD-EPI equation
| CKD-EPI Equation | | |
| --- | --- | --- |
| Sex | Female | Male |
| Age | 43 years | |
| Serum Creatinine | 3 mg/mL | |
| Race | Black | Non-black |
| 21 mL/min/1.73 m² | Stage IV CKD | |
| CKD-EPI Equation | | |
| --- | --- | --- |
| Sex | Female | Male |
| Age | 43 years | |
| Serum Creatinine | 3 mg/mL | |
| Race | Black | Non-black |
| 18 mL/min/1.73 m² | Stage IV CKD | |
Black
Non-Black
4Levey et al., 2009

## Slide 21
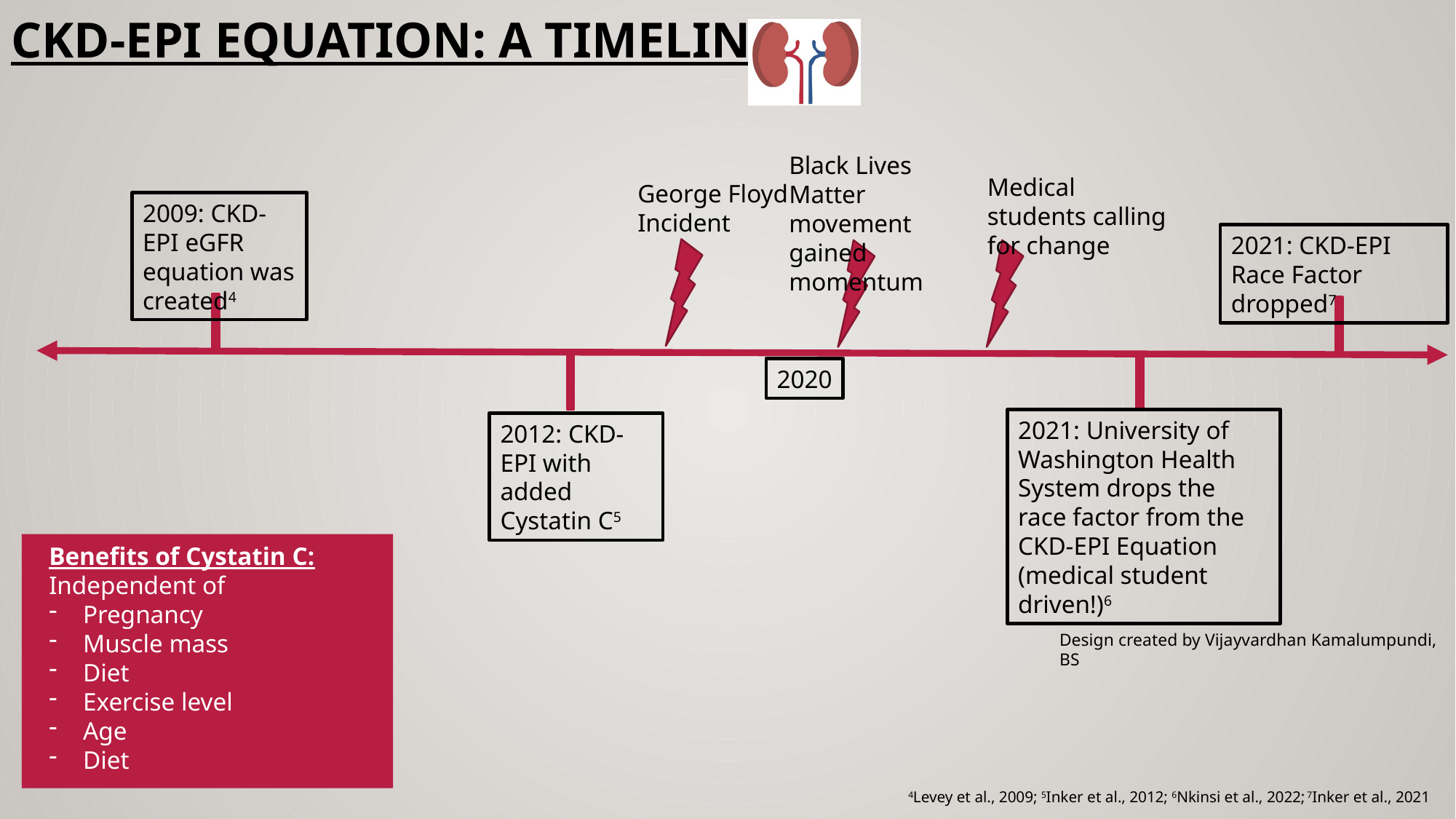

CKD-EPI equation: a timeline
Black Lives Matter movement gained momentum
Medical students calling for change
George Floyd Incident
2009: CKD-EPI eGFR equation was created4
2021: CKD-EPI Race Factor dropped7
2020
2021: University of Washington Health System drops the race factor from the CKD-EPI Equation (medical student driven!)6
2012: CKD-EPI with added Cystatin C5
Benefits of Cystatin C:
Independent of
Pregnancy
Muscle mass
Diet
Exercise level
Age
Diet
Design created by Vijayvardhan Kamalumpundi, BS
4Levey et al., 2009; 5Inker et al., 2012; 6Nkinsi et al., 2022; 7Inker et al., 2021

## Slide 22
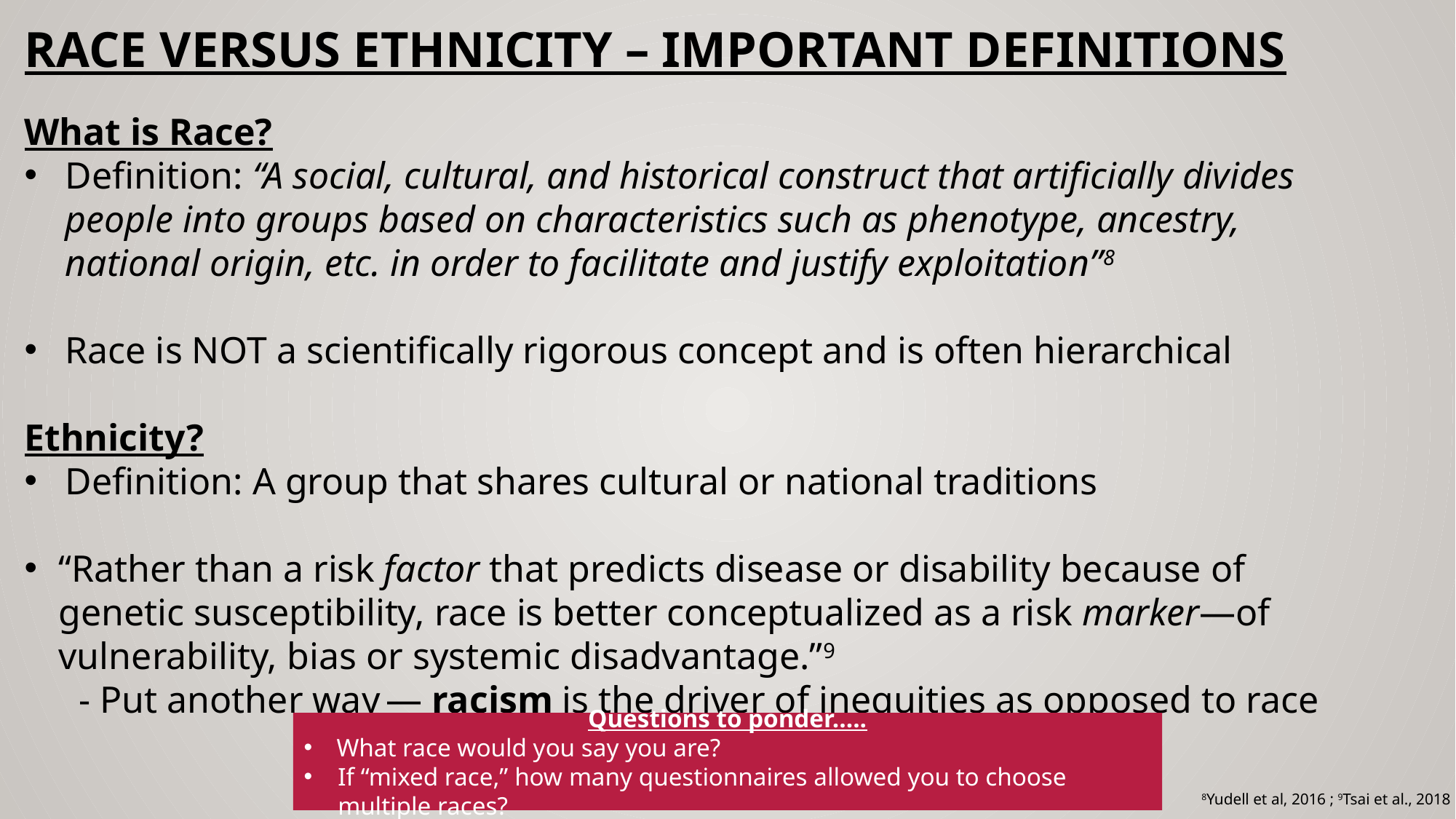

Race VERSUS Ethnicity – Important DEFINITIONS
What is Race?
Definition: “A social, cultural, and historical construct that artificially divides people into groups based on characteristics such as phenotype, ancestry, national origin, etc. in order to facilitate and justify exploitation”8
Race is NOT a scientifically rigorous concept and is often hierarchical
Ethnicity?
Definition: A group that shares cultural or national traditions
“Rather than a risk factor that predicts disease or disability because of genetic susceptibility, race is better conceptualized as a risk marker—of vulnerability, bias or systemic disadvantage.”9
- Put another way — racism is the driver of inequities as opposed to race
Questions to ponder…..
 What race would you say you are?
If “mixed race,” how many questionnaires allowed you to choose multiple races?
8Yudell et al, 2016 ; 9Tsai et al., 2018

## Slide 23
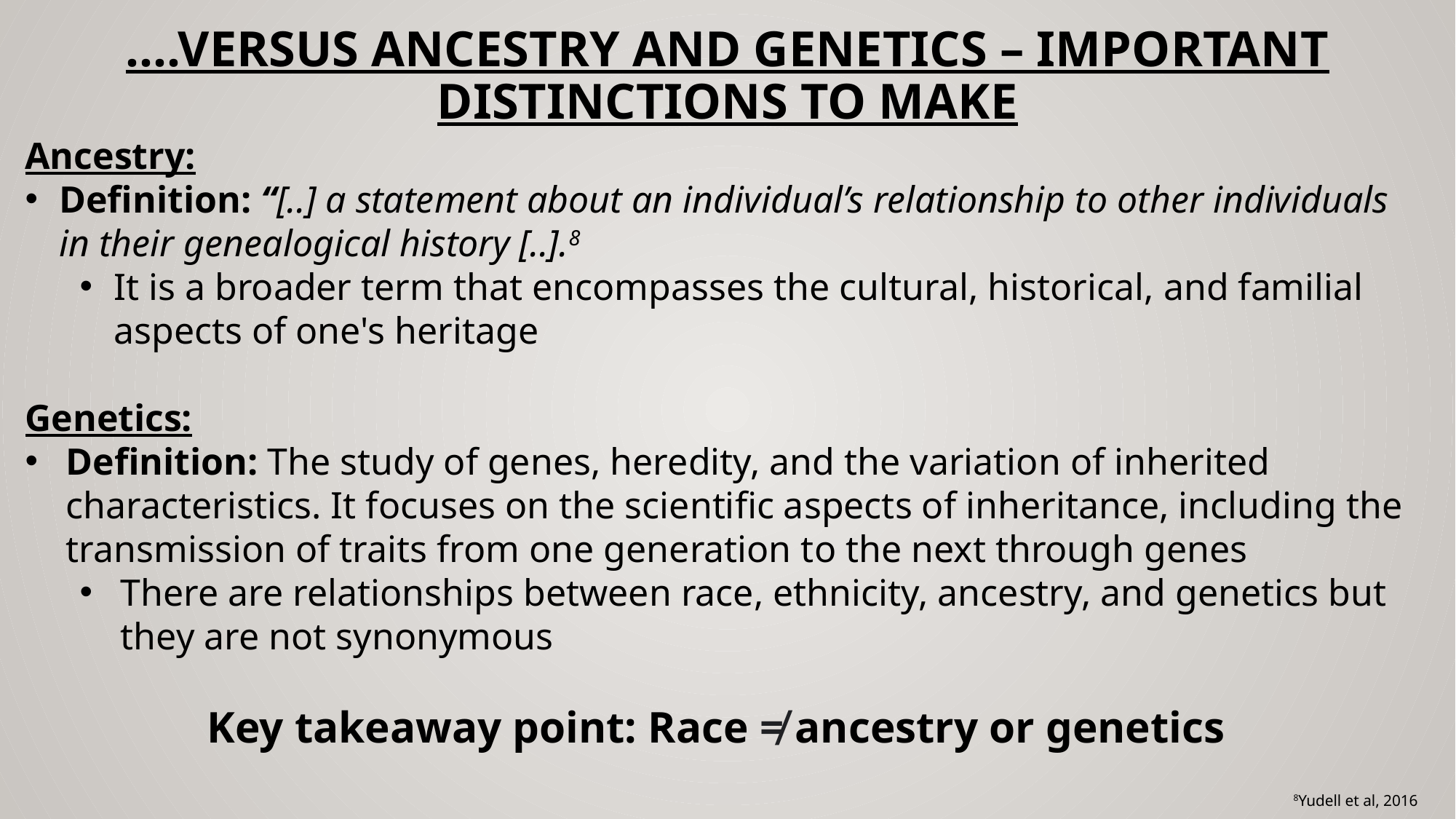

….Versus Ancestry and genetics – important distinctions to make
Ancestry:
Definition: “[..] a statement about an individual’s relationship to other individuals in their genealogical history [..].8
It is a broader term that encompasses the cultural, historical, and familial aspects of one's heritage
Genetics:
Definition: The study of genes, heredity, and the variation of inherited characteristics. It focuses on the scientific aspects of inheritance, including the transmission of traits from one generation to the next through genes
There are relationships between race, ethnicity, ancestry, and genetics but they are not synonymous
Key takeaway point: Race ≠ ancestry or genetics
8Yudell et al, 2016

## Slide 24
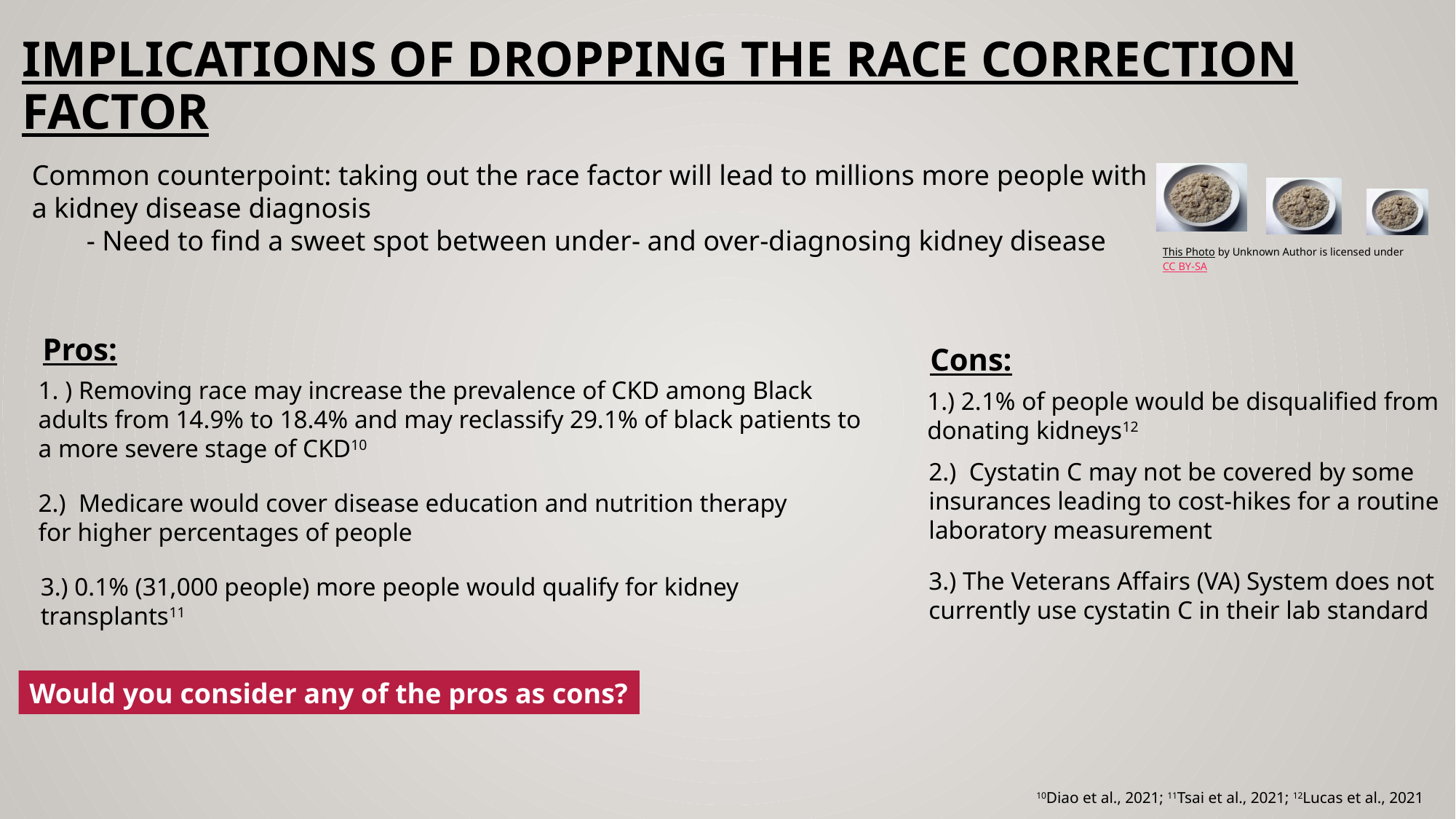

Implications of dropping the race correction factor
Common counterpoint: taking out the race factor will lead to millions more people with a kidney disease diagnosis
	- Need to find a sweet spot between under- and over-diagnosing kidney disease
This Photo by Unknown Author is licensed under CC BY-SA
Pros:
Cons:
1. ) Removing race may increase the prevalence of CKD among Black adults from 14.9% to 18.4% and may reclassify 29.1% of black patients to a more severe stage of CKD10
1.) 2.1% of people would be disqualified from donating kidneys12
2.) Cystatin C may not be covered by some insurances leading to cost-hikes for a routine laboratory measurement
2.) Medicare would cover disease education and nutrition therapy for higher percentages of people
3.) The Veterans Affairs (VA) System does not currently use cystatin C in their lab standard
3.) 0.1% (31,000 people) more people would qualify for kidney transplants11
Would you consider any of the pros as cons?
10Diao et al., 2021; 11Tsai et al., 2021; 12Lucas et al., 2021

## Slide 25
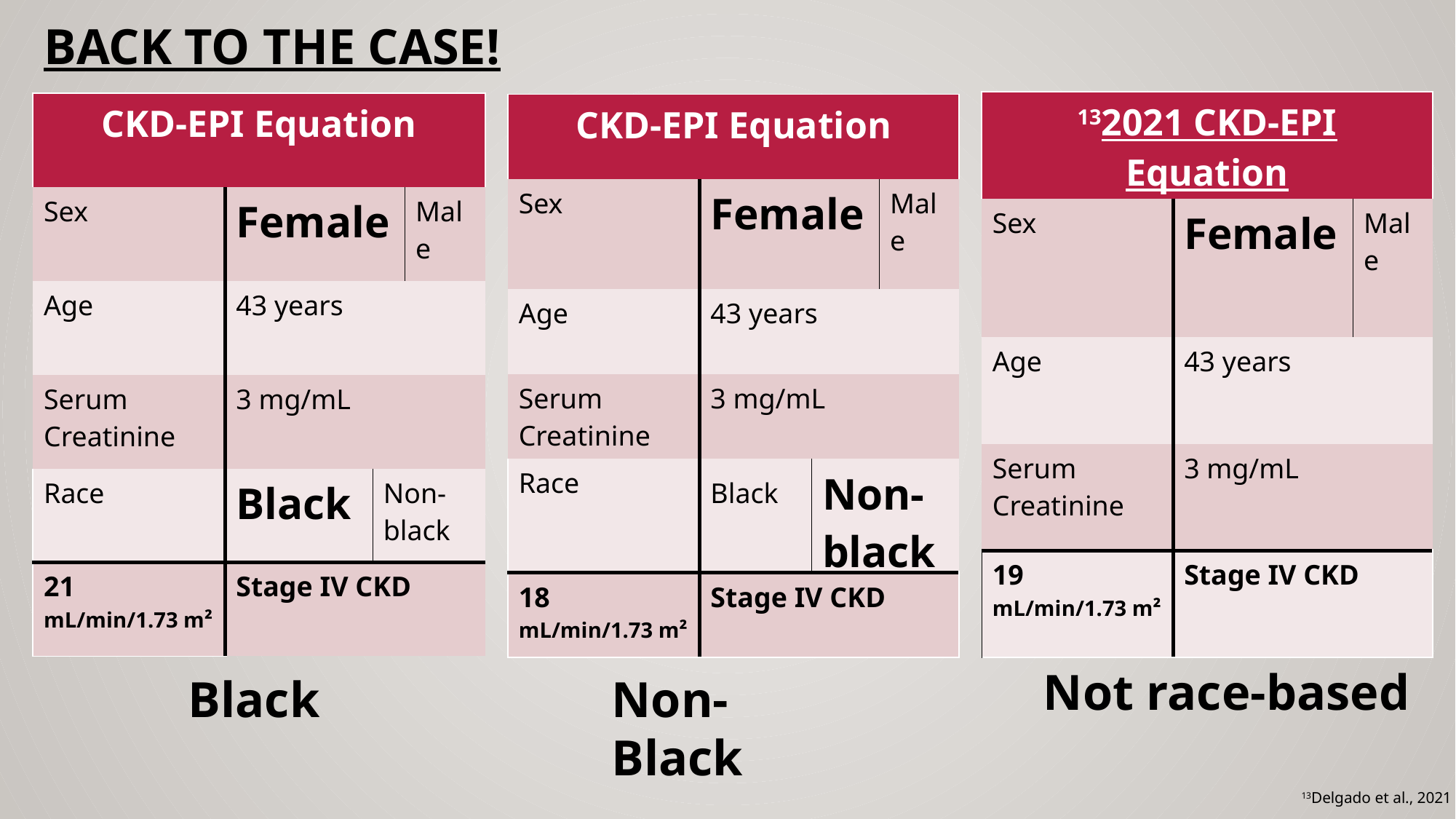

Back to the case!
| 132021 CKD-EPI Equation | | |
| --- | --- | --- |
| Sex | Female | Male |
| Age | 43 years | |
| Serum Creatinine | 3 mg/mL | |
| 19 mL/min/1.73 m² | Stage IV CKD | |
| CKD-EPI Equation | | | |
| --- | --- | --- | --- |
| Sex | Female | Male | Male |
| Age | 43 years | | |
| Serum Creatinine | 3 mg/mL | | |
| Race | Black | Non-black | |
| 21 mL/min/1.73 m² | Stage IV CKD | | |
| CKD-EPI Equation | | | |
| --- | --- | --- | --- |
| Sex | Female | | Male |
| Age | 43 years | | |
| Serum Creatinine | 3 mg/mL | | |
| Race | Black | Non-black | Non-black |
| 18 mL/min/1.73 m² | Stage IV CKD | | |
Not race-based
Black
Non-Black
13Delgado et al., 2021

## Slide 26
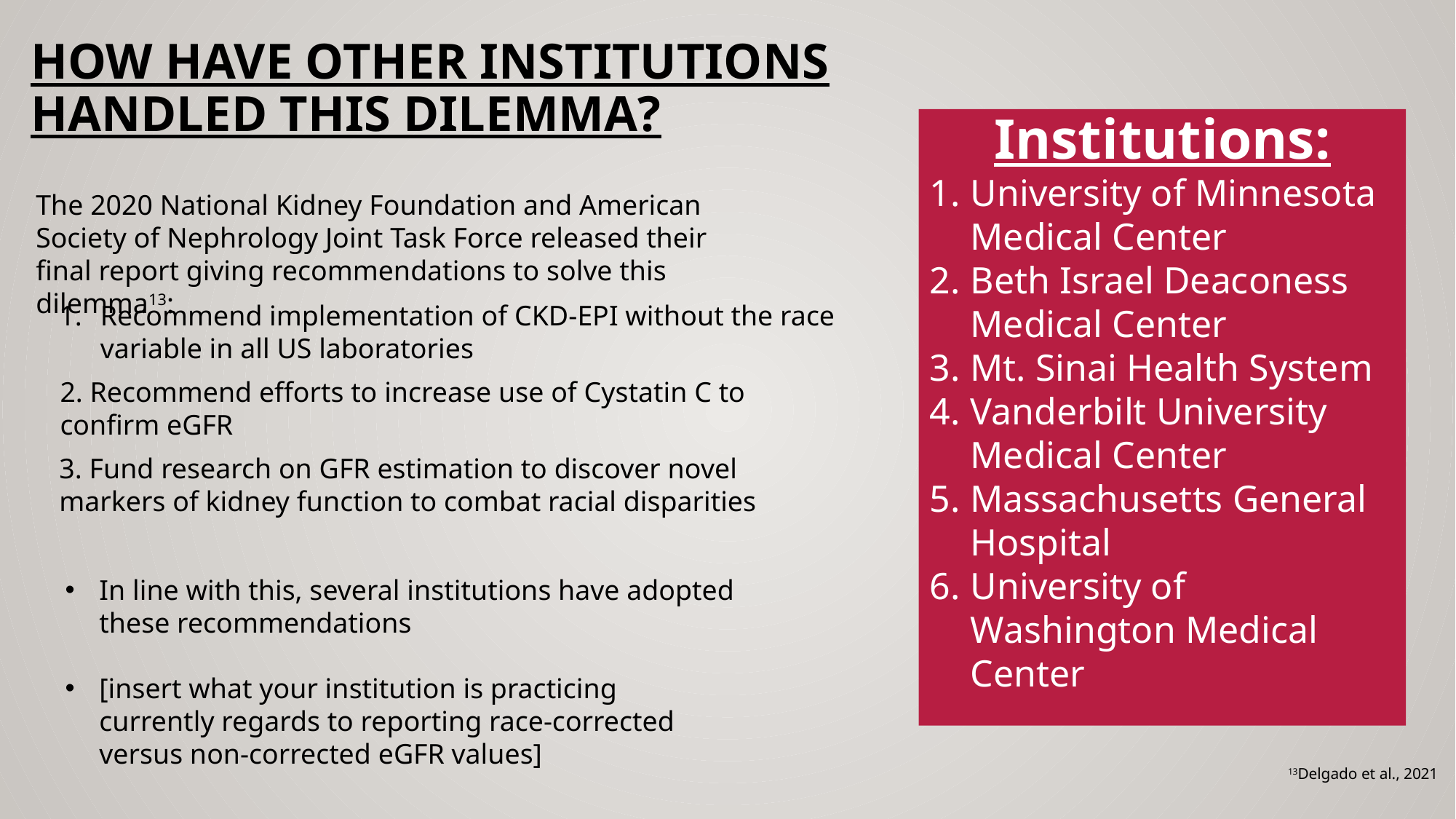

How have other institutions handled this dilemma?
Institutions:
University of Minnesota Medical Center
Beth Israel Deaconess Medical Center
Mt. Sinai Health System
Vanderbilt University Medical Center
Massachusetts General Hospital
University of Washington Medical Center
The 2020 National Kidney Foundation and American Society of Nephrology Joint Task Force released their final report giving recommendations to solve this dilemma13:
Recommend implementation of CKD-EPI without the race variable in all US laboratories
2. Recommend efforts to increase use of Cystatin C to confirm eGFR
3. Fund research on GFR estimation to discover novel markers of kidney function to combat racial disparities
In line with this, several institutions have adopted these recommendations
[insert what your institution is practicing currently regards to reporting race-corrected versus non-corrected eGFR values]
13Delgado et al., 2021

## Slide 27
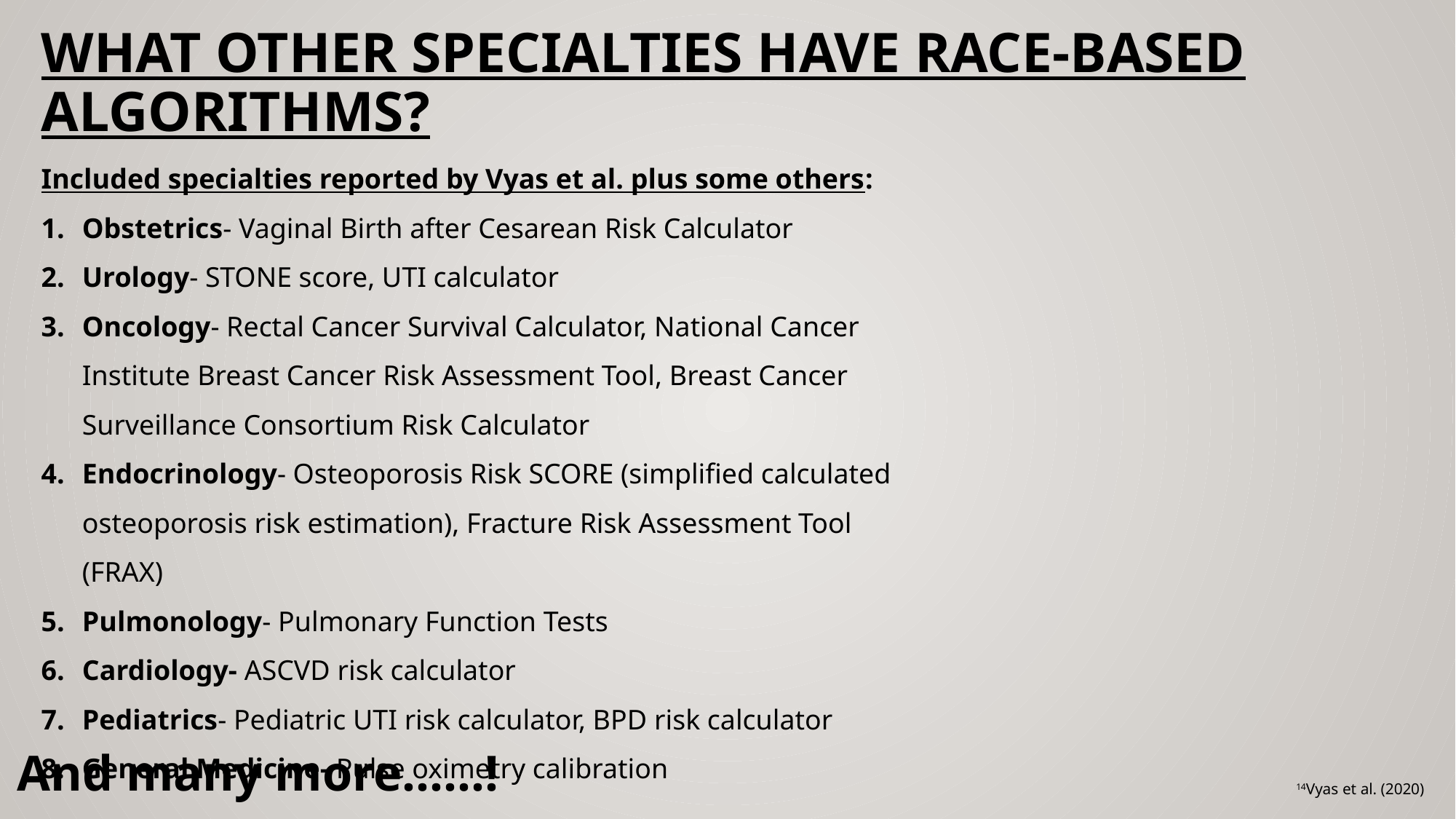

What other specialties have race-based algorithms?
Included specialties reported by Vyas et al. plus some others:
Obstetrics- Vaginal Birth after Cesarean Risk Calculator
Urology- STONE score, UTI calculator
Oncology- Rectal Cancer Survival Calculator, National Cancer Institute Breast Cancer Risk Assessment Tool, Breast Cancer Surveillance Consortium Risk Calculator
Endocrinology- Osteoporosis Risk SCORE (simplified calculated osteoporosis risk estimation), Fracture Risk Assessment Tool (FRAX)
Pulmonology- Pulmonary Function Tests
Cardiology- ASCVD risk calculator
Pediatrics- Pediatric UTI risk calculator, BPD risk calculator
General Medicine- Pulse oximetry calibration
And many more……!
14Vyas et al. (2020)

## Slide 28
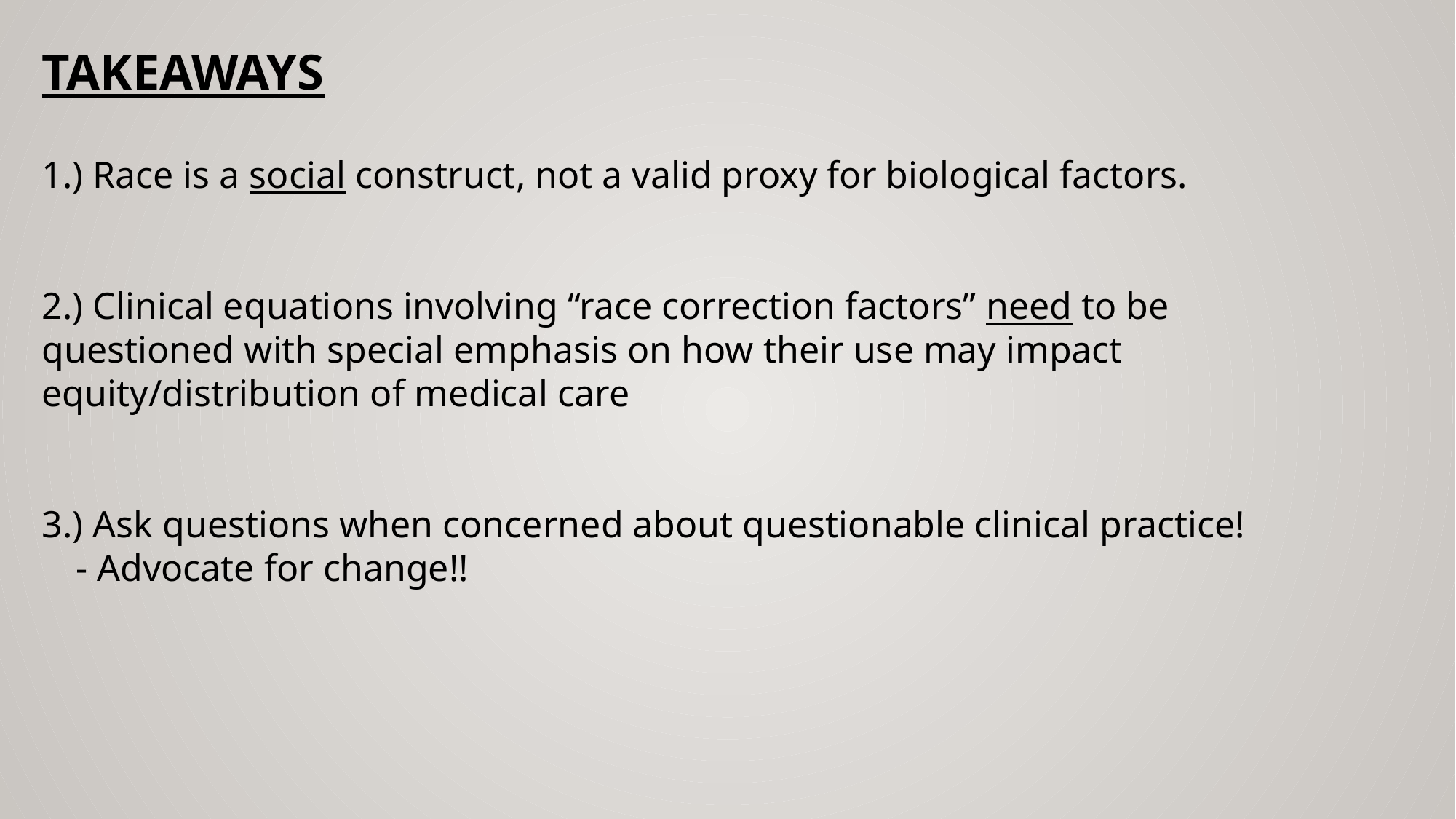

Takeaways
1.) Race is a social construct, not a valid proxy for biological factors.
2.) Clinical equations involving “race correction factors” need to be questioned with special emphasis on how their use may impact equity/distribution of medical care
3.) Ask questions when concerned about questionable clinical practice!
- Advocate for change!!

## Slide 29
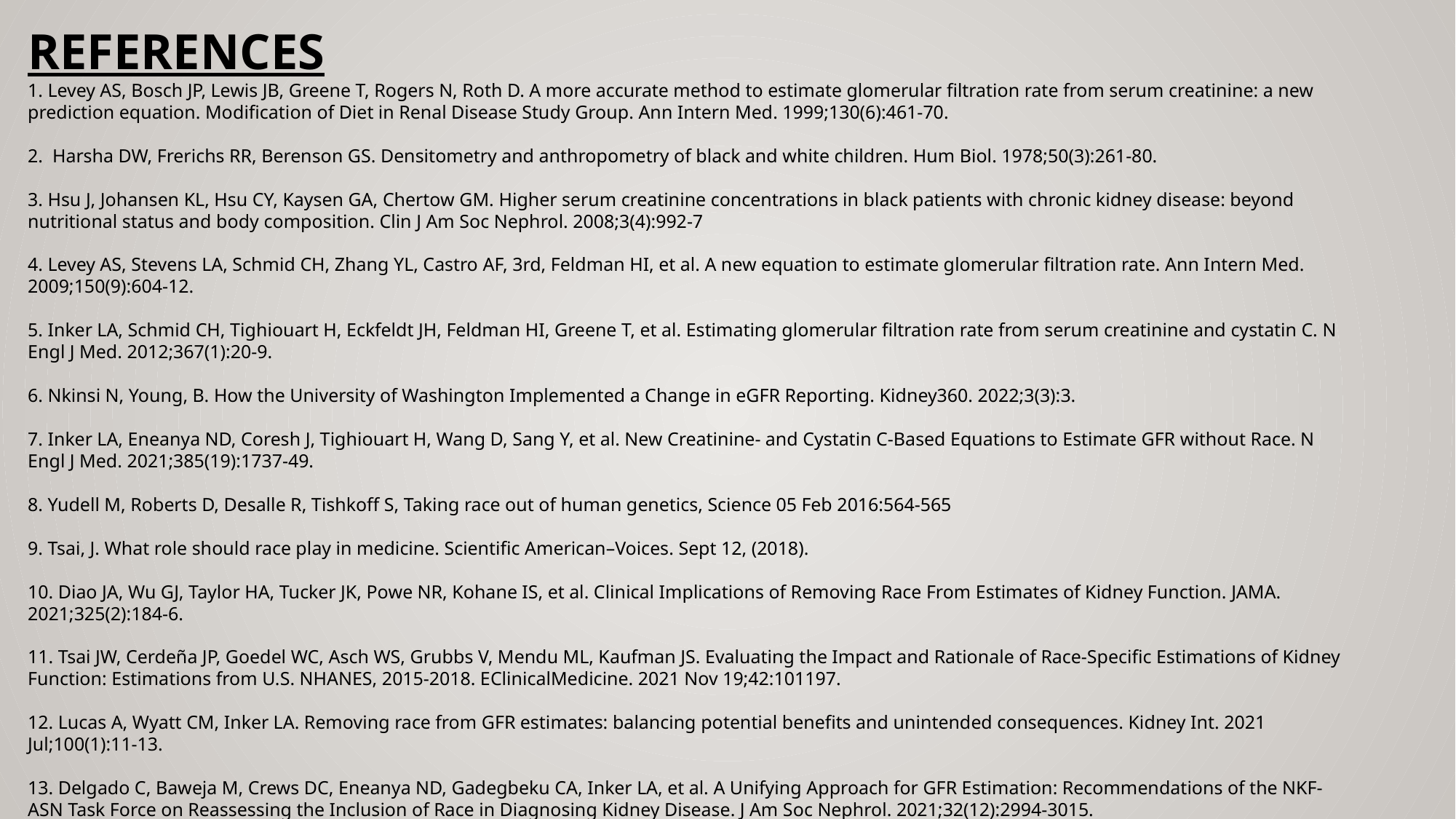

References
1. Levey AS, Bosch JP, Lewis JB, Greene T, Rogers N, Roth D. A more accurate method to estimate glomerular filtration rate from serum creatinine: a new prediction equation. Modification of Diet in Renal Disease Study Group. Ann Intern Med. 1999;130(6):461-70.
2. Harsha DW, Frerichs RR, Berenson GS. Densitometry and anthropometry of black and white children. Hum Biol. 1978;50(3):261-80.
3. Hsu J, Johansen KL, Hsu CY, Kaysen GA, Chertow GM. Higher serum creatinine concentrations in black patients with chronic kidney disease: beyond nutritional status and body composition. Clin J Am Soc Nephrol. 2008;3(4):992-7
4. Levey AS, Stevens LA, Schmid CH, Zhang YL, Castro AF, 3rd, Feldman HI, et al. A new equation to estimate glomerular filtration rate. Ann Intern Med. 2009;150(9):604-12.
5. Inker LA, Schmid CH, Tighiouart H, Eckfeldt JH, Feldman HI, Greene T, et al. Estimating glomerular filtration rate from serum creatinine and cystatin C. N Engl J Med. 2012;367(1):20-9.
6. Nkinsi N, Young, B. How the University of Washington Implemented a Change in eGFR Reporting. Kidney360. 2022;3(3):3.
7. Inker LA, Eneanya ND, Coresh J, Tighiouart H, Wang D, Sang Y, et al. New Creatinine- and Cystatin C-Based Equations to Estimate GFR without Race. N Engl J Med. 2021;385(19):1737-49.
8. Yudell M, Roberts D, Desalle R, Tishkoff S, Taking race out of human genetics, Science 05 Feb 2016:564-565
9. Tsai, J. What role should race play in medicine. Scientific American–Voices. Sept 12, (2018).
10. Diao JA, Wu GJ, Taylor HA, Tucker JK, Powe NR, Kohane IS, et al. Clinical Implications of Removing Race From Estimates of Kidney Function. JAMA. 2021;325(2):184-6.
11. Tsai JW, Cerdeña JP, Goedel WC, Asch WS, Grubbs V, Mendu ML, Kaufman JS. Evaluating the Impact and Rationale of Race-Specific Estimations of Kidney Function: Estimations from U.S. NHANES, 2015-2018. EClinicalMedicine. 2021 Nov 19;42:101197.
12. Lucas A, Wyatt CM, Inker LA. Removing race from GFR estimates: balancing potential benefits and unintended consequences. Kidney Int. 2021 Jul;100(1):11-13.
13. Delgado C, Baweja M, Crews DC, Eneanya ND, Gadegbeku CA, Inker LA, et al. A Unifying Approach for GFR Estimation: Recommendations of the NKF-ASN Task Force on Reassessing the Inclusion of Race in Diagnosing Kidney Disease. J Am Soc Nephrol. 2021;32(12):2994-3015.
14. Vyas DA, Eisenstein LG, Jones DS. Hidden in Plain Sight - Reconsidering the Use of Race Correction in Clinical Algorithms. N Engl J Med. 2020;383(9):874-82.

## Slide 30
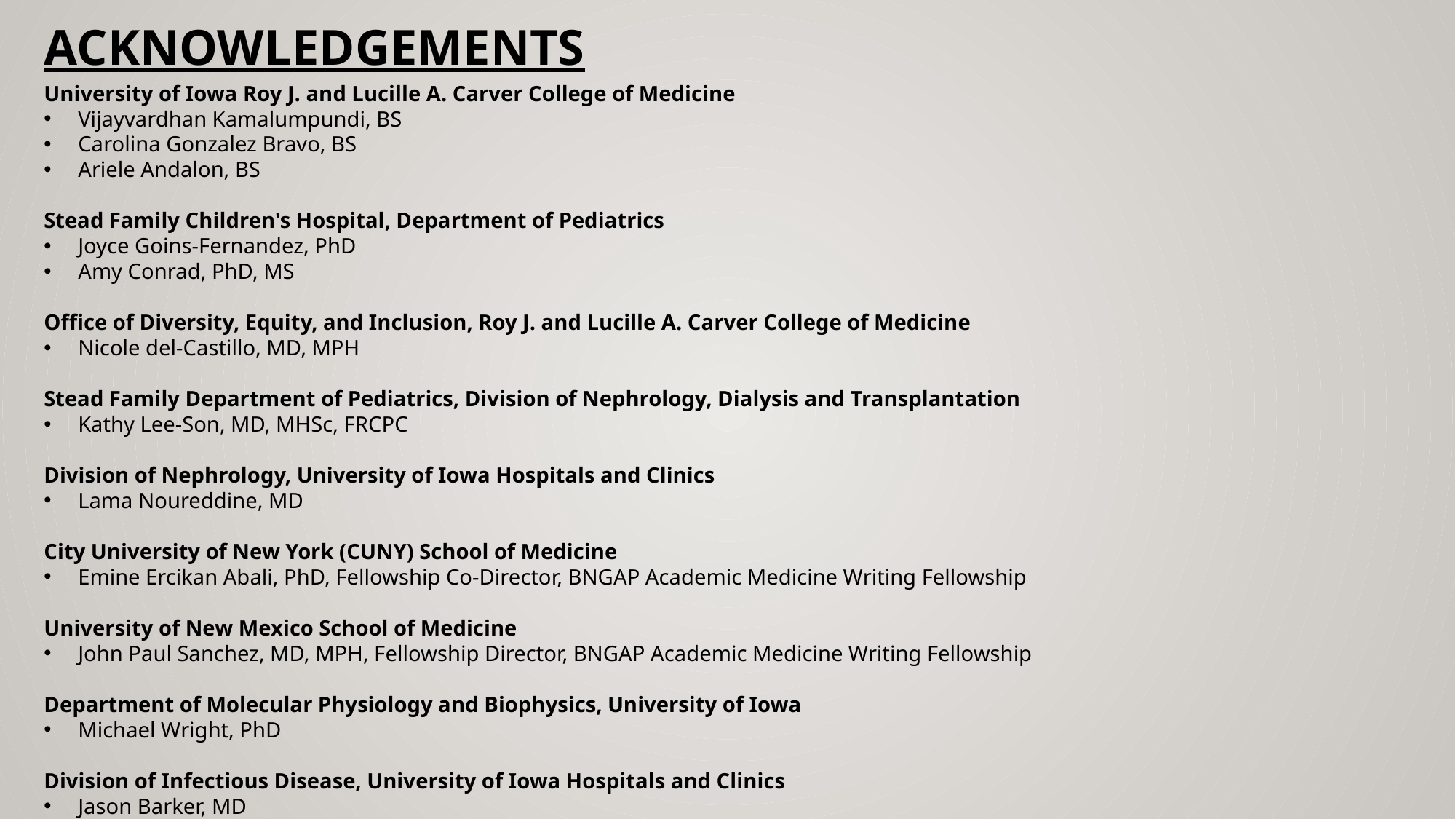

Acknowledgements
University of Iowa Roy J. and Lucille A. Carver College of Medicine
Vijayvardhan Kamalumpundi, BS
Carolina Gonzalez Bravo, BS
Ariele Andalon, BS
Stead Family Children's Hospital, Department of Pediatrics
Joyce Goins-Fernandez, PhD
Amy Conrad, PhD, MS
Office of Diversity, Equity, and Inclusion, Roy J. and Lucille A. Carver College of Medicine
Nicole del-Castillo, MD, MPH
Stead Family Department of Pediatrics, Division of Nephrology, Dialysis and Transplantation
Kathy Lee-Son, MD, MHSc, FRCPC
Division of Nephrology, University of Iowa Hospitals and Clinics
Lama Noureddine, MD
City University of New York (CUNY) School of Medicine
Emine Ercikan Abali, PhD, Fellowship Co-Director, BNGAP Academic Medicine Writing Fellowship
University of New Mexico School of Medicine
John Paul Sanchez, MD, MPH, Fellowship Director, BNGAP Academic Medicine Writing Fellowship
Department of Molecular Physiology and Biophysics, University of Iowa
Michael Wright, PhD
Division of Infectious Disease, University of Iowa Hospitals and Clinics
Jason Barker, MD

## Slide 31
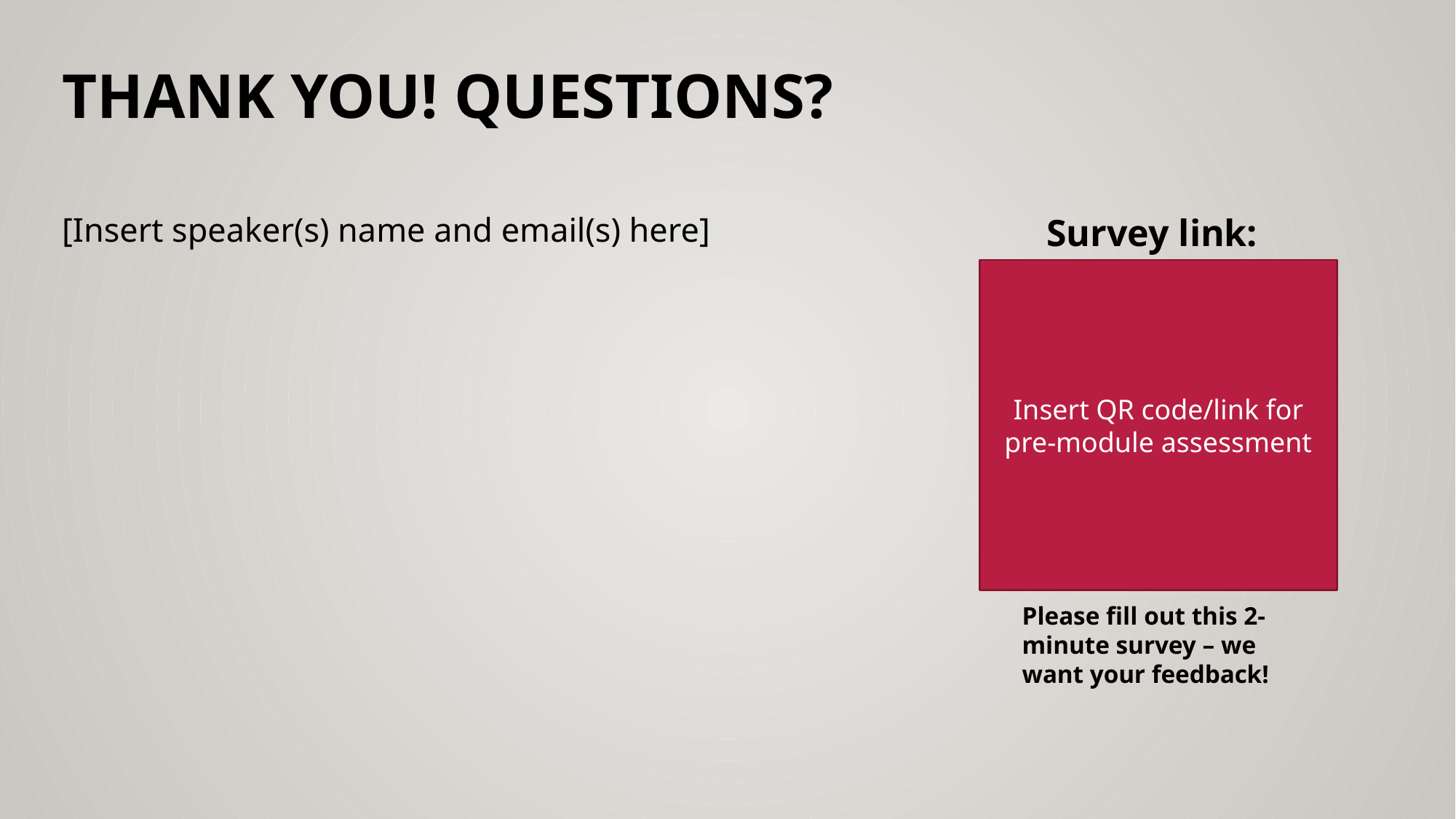

Thank you! Questions?
Survey link:
[Insert speaker(s) name and email(s) here]
Insert QR code/link for pre-module assessment
Please fill out this 2-minute survey – we want your feedback!
